# Complete, Theoretical Rovibronic Spectral Characterization of the Carbon Monoxide, Water, and Formaldehyde Cations

**DOI:** 10.3390/molecules28041782

**Published:** 2023-02-13

**Authors:** Megan C. Davis, Xinchuan Huang, Ryan C. Fortenberry

**Affiliations:** 1 Department of Chemistry & Biochemistry, University of Mississippi, University, MS 38677-1848, USA; 2 SETI Institute, Mountain View, CA 94043, USA; 3 MS 245-6, NASA Ames Research Center, Moffett Field, CA 94035, USA

**Keywords:** quantum chemistry, computational spectroscopy, coupled cluster theory, astrochemistry, UV/Vis spectra, rovibronic spectra

## Abstract

New high-level ab initio quartic force field (QFF) methods are explored which provide spectroscopic data for the electronically excited states of the carbon monoxide, water, and formaldehyde cations, sentinel species for expanded, recent cometary spectral analysis. QFFs based on equation-of-motion ionization potential (EOM-IP) with a complete basis set extrapolation and core correlation corrections provide assignment for the fundamental vibrational frequencies of the $\tilde{A}$ ${}^{2}$B${}_{1}$ and $\tilde{B}$ ${}^{2}$A${}_{1}$ states of the formaldehyde cation; only three of these frequencies have experimental assignment available. Rotational constants corresponding to these vibrational excitations are also provided for the first time for all electronically excited states of both of these molecules. EOM-IP-CCSDT/CcC computations support tentative re-assignment of the $\nu_{1}$ and $\nu_{3}$ frequencies of the $\tilde{B}$ ${}^{2}B_{2}$ state of the water cation to approximately 2409.3 cm${}^{-1}$ and 1785.7 cm${}^{-1}$, respectively, due to significant disagreement between experimental assignment and all levels of theory computed herein, as well as work by previous authors. The EOM-IP-CCSDT/CcC QFF achieves agreement to within 12 cm${}^{-1}$ for the fundamental vibrational frequencies of the electronic ground state of the water cation compared to experimental values and to the high-level theoretical benchmarks for variationally-accessible states. Less costly EOM-IP based approaches are also explored using approximate triples coupled cluster methods, as well as electronically excited state QFFs based on EOM-CC3 and the previous (T)+EOM approach. The novel data, including vibrationally corrected rotational constants for all states studied herein, provided by these computations should be useful in clarifying comet evolution or other remote sensing applications in addition to fundamental spectroscopy.

## 1. Introduction

Small molecular cations would be potent markers for comet evolution in the Solar System, as these species are believed to be abundant in cometary comae [1]. Understanding the rovibronic transitions of these small molecules would aid in their potential inclusion as markers of photochemical processes in these environments [2]. Previously unidentified lines in the spectra of the Hyakutake and Ikeya–Zhang comets [3] have been attributed to transitions of higher-excited levels of H${}_{2}$O${}^{+}$ [4], exemplifying the importance of these rovibronic transitions. The “ROSINA ion zoo” observed by the Rosetta spacecraft at comet 67P/Churyumov–Gerasimenko underscores the importance of these species as sentinels for important astrophysical phenomena [5] through in situ mass spectroscopic detection of many small cation species such as CH${}_{3}$ ${}^{+}$, H${}_{2}$O${}^{+}$, HCO${}^{+}$ and CH${}_{3}$OH${}_{2}$ ${}^{+}$. Additionally, characterization of the rovibronic and photoionization spectra of such species would help connect unassigned spectral lines with these transitions, thereby “pulling the weeds” of spectra observed near various Solar System bodies. This would indirectly aid in the detection of new molecules by clarifying unknown spectra of known molecules. Many cometary lines from the near-UV and visible regions of the electromagnetic spectrum are unattributed, representing significant knowledge gaps in the characterization of these bodies and their rovibronic spectra [3,6,7,8,9,10,11,12,13]. The detection of H${}_{2}$O${}^{+}$ and CO${}^{+}$ in situ by the Rosetta spacecraft as well as the presence of peaks in low resolution double focusing mass spectrometer results corresponding to the mass of H${}_{2}$CO${}^{+}$, also during this mission to comet 67P, make an understanding of the full rovibronic spectra of these three molecules desirable for subsequent remote sensing within planetary science beyond fundamental spectroscopic clarification of these three molecular cations.

While some experimental rovibronic spectral data are known for these molecules, many constants are still missing. Modern quantum chemical analysis can provide a complete set of such data. However, no good means for quantum chemically computing anharmonic vibrational parameters of electronically excited states exist. This is necessary to produce a full set of vibrationally-corrected rotational constants for each electronic state. Although this is not all that is necessary for a complete model of electronic spectra, these data are a portion of what is required for a complete UV/Vis model of these electronic excitations.

Looking at the above set of small molecular cations, CO${}^{+}$ has already been extensively explored with theoretical approaches [14,15,16] and many of its spectroscopic constants are well characterized experimentally [17,18]. However, because it is a small system similar to H${}_{2}$O${}^{+}$ and H${}_{2}$CO${}^{+}$, it should act as a reasonable benchmark for electronically excited state methods that may then be applied to the latter molecules. Thus, rovibrationally probing the electronically excited states of CO${}^{+}$ with the methods outlined in the present work will validate the utility of said methods for small molecular radical cations as well as producing useful and novel spectroscopic data for CO${}^{+}$, such as predictions for the H${}_{e}$ distortion constant. The $\tilde{X}$ ${}^{2}\Sigma^{+}$, $\tilde{A}$ ${}^{2}\Pi$, and $\tilde{B}$ ${}^{2}\Sigma^{+}$ states of CO${}^{+}$, i.e. the lowest-lying states, are the primary electronic states of astrochemical interest and will be explored in the present study.

H${}_{2}$O${}^{+}$ and H${}_{2}$CO${}^{+}$ have several stable, low-lying excited electronic states, showcasing these molecules as prime candidates for rovibronic characterization. These excited electronic states of H${}_{2}$O${}^{+}$ have been explored quite extensively experimentally [19,20,21,22,23,24,25] and theoretically [26,27]. Similarly, H${}_{2}$CO${}^{+}$ has also been explored with both experiment and theory [28,29,30,31,32]. However, significant gaps still remain for their spectral classification [2].

H${}_{2}$O${}^{+}$ has a stable $\tilde{A}$ ${}^{2}$A${}_{1}$ state and a meta-stable $\tilde{B}$ ${}^{2}$B${}_{2}$ state [33]. The $\tilde{A}$ ${}^{2}$A${}_{1}$ state of H${}_{2}$O${}^{+}$ is linear and exhibits classic Renner–Teller behavior [34]. Its equilibrium geometry is linear, at which point the species is properly labeled as a Σ state but is degenerate with the $\tilde{X}$ ${}^{2}$B${}_{1}$ state at linearity. The equilibrium geometry of the $\tilde{B}$ ${}^{2}$B${}_{2}$ state is, however, highly bent to even less than 60${}^{\circ }$. The $\tilde{B}$ ${}^{2}$B${}_{2}$ state has complex observed spectra due to coupling with the $\tilde{X}$ and $\tilde{A}$ surfaces [20,27] most notably in the form of a conical intersection between the $\tilde{A}$ ${}^{2}$A${}_{1}$ and $\tilde{B}$ ${}^{2}$B${}_{2}$ states [26]. Consequently, these species have been treated in the past with multi-reference methods [35] as well as diabatic treatments [27]. However, there is considerable practical difficulty with such approaches, where the use of more simplified methods would be desired, if possible, in order to provide additional data. Another complication is that the low intensity transitions of the lower vibrational excitations of the $\tilde{A}$ ${}^{2}$A${}_{1}$ state [23,35] result in poor experimental characterization of this state. As a result, further analysis of these transitions with other methods is warranted.

H${}_{2}$CO${}^{+}$ has transitions into the $\tilde{A}$ ${}^{2}$B${}_{1}$ and $\tilde{B}$ ${}^{2}$A${}_{1}$ states in the visible range, which share a similar planar geometry with the ground electronic state [31]. Characterization of these states is relatively more straightforward than the H${}_{2}$O${}^{+}$ system, although many vibrational modes have not been experimentally characterized. This represents an opportunity for high-level theoretical spectroscopy to provide novel insights.

Even though experimental and theoretical spectroscopic data are available for several of these species, there is significant room for improvement in their characterization. Most experimental data come from photoelectron spectra, where there is difficulty in accurate assignment, and some data are of fairly low resolution [20,28,29,36,37]. For instance, Feller and Davidson [38] argue for a reassignment of the fundamental vibrational frequencies of the $\tilde{B}$ ${}^{2}$B${}_{2}$ state of H${}_{2}$O${}^{+}$ attributed from photoelectron spectra. The highly accurate ab initio adiabatic ionization potentials computed in [38] imply that the fundamental frequencies are likely mislabeled, highlighting the role that theory can play in clarifying the spectra of such species beyond providing corroboration with purely experimental inferences. Additionally, providing theoretical data for low intensity vibrational frequencies would assist in thermodynamic characterization of these species and complete spectral modeling as would be required for the classification of cometary observations. Herein, a simple, but highly accurate [39] adiabatic approach is utilized in an attempt to provide fundamental vibrational frequencies for the electronically excited states for CO${}^{+}$, H${}_{2}$O${}^{+}$ and H${}_{2}$CO${}^{+}$.

Quartic force fields (QFFs) can be used readily and accurately to characterize vibrational frequencies, rotational constants, and other spectroscopic parameters for molecules in their ground electronic states [40]. These have previously been extended to electronically excited states [41,42] using EOM-CC3 [43,44] as well as the recently proposed (T)+EOM approach [39]. The last method has achieved mean absolute differences as low as 1.6 cm${}^{-1}$ for anharmonic frequencies relative to the established benchmark CcCR [45] approach (defined below). Although the (T)+EOM method seems promising, it has not yet been applied beyond the initial set of test cases. Thus, formulating alternative electronically excited state QFF approaches is necessary in order to produce accurate high-level spectroscopic data for these small molecular cations for assisting in their role as markers of cometary phenomenon.

In this work, the application of quartic force fields based on the ionization potential variant of equation-of-motion (EOM-IP) [46] is undertaken. This allows for usage of a closed-shell reference state to describe the open-shell target electronic states. These closed-shell references are in many cases more well-behaved than their open-shell counterparts, making such a treatment less prone to computational pitfalls. Additionally, EOM-IP based approaches allow for a broader choice of quantum chemical codes, which account for higher order correlation since many excited state methods are not implemented for open-shell references. The application of EOM-IP to these systems is not wholly novel [14]; however, the QFFs explored in the present study provide anharmonic data at a higher level than has been previously available. These QFF approaches may also be useful for astrochemical applications beyond the present study. Beyond EOM-IP, the straightforward equation-of-motion excitation energy (EOM-EE) based QFFs [47] are also employed to provide potentially corroborating data for the molecules of interest if the reference states are sufficiently well-behaved. EOM-EE-CC3 is one of the only methods with higher order correlation widely available for open-shell references [44], and it forms the basis for one set of the QFFs employed here.

Thus, EOM-IP based QFFs together with (T)+EOM are used to explore low-lying excited electronic states of CO${}^{+}$, H${}_{2}$CO${}^{+}$ and H${}_{2}$O${}^{+}$, with the intention of providing what is necessary for a complete theoretical model of these species’ spectra in the UV/Vis region. Together with other forms of characterization beyond the scope of the present study (e.g., oscillator strengths), the data presented herein should be fruitful for photophysical applications such as the study of cometary phenomena, especially upon perhelion.

## 2. Computational Methods

Spectroscopic data for ground state species are computed with two primary QFF approaches. The first QFF uses explicitly correlated coupled cluster singles, doubles, and perturbative triples energies within the F12 formalism [CCSD(T)-F12b] [48] conjoined to the cc-pVTZ-F12 basis set [49,50,51]. This is dubbed the F12-TZ approach from here on [52,53]. The second uses energies consisting of a three-point aug-cc-pVXZ (X = T, Q, 5) complete basis set extrapolation (CBS: C) [54] with additive core correlation (cC) corrections using the Martin–Taylor basis set [55] and an additional scalar relativistic correction using the Douglas–Kroll (R) formalism [56]: this is known as the CcCR approach [45]. The CBS extrapolation used is given by the following formula, where *A*, *B*, and *C* are the 5Z, QZ, and TZ basis set computations, respectively, and *l* is the highest angular momentum included in the given basis set:

(1) $$E\left(l\right)=A+B{\left(l+1/2\right)}^{-4}+C{\left(l+1/2\right)}^{-6}.$$

For other QFFs which use a two-point extrapolation, the following formula is used:

(2) $$E\left(l\right)=A+Bl^{-3}.$$

The Douglas–Kroll correction is computed at the CCSD(T) level using a triple-zeta Douglas–Kroll basis set [57] as the difference between the energy computed with relativity enabled and disabled. For F12-TZ, optimized reference geometries are obtained using CCSD(T)-F12b/cc-pVTZ-F12 energies. CcCR uses reference geometries obtained at the CCSD(T)/aug-cc-pV5Z level corrected by a Martin–Taylor core correlation correction. All computations for the CcCR and F12-TZ QFFs are performed using MOLPRO 2020 [58].

The QFF is formed by displacing the reference geometry at step sizes of 0.005 Å or radians along a set of symmetry internal coordinates (SICs). The SIC schemes employed for each species are given in Appendix A. The single point energies described above are computed at each of the displaced geometries. Force constants are then generated by performing a least squares fitting of these energies followed by a refitting to the numerically exact minimum geometry. The resulting force constants of the refit are then converted to Cartesian coordinates by the INTDER program [59]. The SPECTRO software package [60] then uses these force constants in second-order vibrational and rotational perturbation theory (VPT2) to produce spectroscopic data [61,62,63].

(Ro)vibrational variational configuration interaction (VCI) calculations are also performed for some QFFs for validation of the VPT2 results. For VCI calculations, the symmetry internal coordinate QFFs are converted to simple internal coordinate QFFs so that α values can be determined [64] for the Morse function of C/O–H and C–O bond stretches. Then, the simple internal coordinate QFFs are analytically converted [65] to simple Morse-cosine(-sin) QFFs, which are adopted in VCI calculations using VTET [66] for H${}_{2}$O${}^{+}$ and MULTIMODE (MM) [67,68] for H${}_{2}$CO${}^{+}$. Later for verification, symmetry Morse-cosine(-sin) QFFs are directly fitted from the same geometry and energy sets of the $\tilde{B}$ ${}^{2}$B${}_{2}$ state of H${}_{2}$O${}^{+}$ and the $\tilde{B}$ ${}^{2}$A${}_{1}$ state of H${}_{2}$CO${}^{+}$, and their MM calculations confirm the reliability and consistency of VCI results acquired on simple Morse-cosine(-sin) QFFs. Tests show the VCI values are converged to better than 0.05 cm${}^{-1}$ (H${}_{2}$O${}^{+}$) or 0.2 cm${}^{-1}$ (H${}_{2}$CO${}^{+}$).

For electronically excited states, (T)+EOM/CcCR QFFs [39] are constructed by approximating the energy of a target higher electronic state as a combination of a ground-state CCSD(T) energy and an EOM-CCSD excitation energy to the target state. These (T)+EOM energies are used with the same core correlation and scalar relativistic corrections as in the ground state CcCR QFF to form the (T)+EOM/CcCR approach. The optimized geometry for the (T)+EOM/CcCR QFF is obtained following the scheme in Equation (3), where $R_{(T)+EOM/CcCR}$ is a given geometric parameter for the final geometry. The scheme for the single point energies ($E_{(T)+EOM/CcCR}$) is shown in Equation (4). The geometry optimizations for the (T)+EOM/CcCR QFFs are constructed in NWChem [69] due to its ability to optimize molecular geometries for the full (T)+EOM energy for open-shell molecules in a straightforward manner without the need for additional, hand-written wrapper programs. The CCSD(T) energies for these QFF points are calculated using MOLPRO 2020.1 [58]. The EOM-CCSD energies are calculated in PSI4 [70] since this program has restricted open-shell Hartree–Fock EOM methods available [44].

(3) $$R_{(T)+EOM/CcCR}\equiv R_{(T)+EOM/aug-cc-pV5Z}+\left(R_{(T)+EOM/MTcore}-R_{(T)+EOM/MT}\right).$$

(4) $$\begin{array}{c} E_{(T)+EOM/CcCR}\equiv E_{(T)+EOM/CBS}+E_{(T)+EOM/MTcore}-E_{(T)+EOM/MT}\\ +E_{(T)+EOM/DKrel}-E_{(T)+EOM/DK}. \end{array}$$

A second electronically excited state approach employed here is based on EOM-EE-CC3. Two approaches are investigated: one uses a two-point CBS extrapolation at the triple-zeta and quadruple-zeta levels (“TQ”); the second uses the same three-point CBS extrapolation (“C”) defined for (T)+EOM/CcCR and CcCR. Both of these levels of theory include an additional core correlation correction term (“cC”) using the Martin–Taylor basis set and are, thus, named EOM-EE-CC3/TQcC and EOM-EE-CC3/CcC, respectively. These computations are performed in PSI4.

Finally, EOM-IP based QFFs are constructed utilizing two iterative perturbative triples methods: EOM-IP-CCSDT-3 and EOM-IP-CC3 [71]. In addition, QFFs at the EOM-IP-CCSDT level are computed. The latter is employed at the EOM-IP-CCSDT/TQcC and EOM-IP-CCSDT/CcC levels, similar to the EOM-EE-CC3 QFFs. EOM-IP-CCSDT-3 and EOM-IP-CC3 employ the same approaches but with an additional corrective term for higher order correlation at the triple zeta level (T):

(5) $$\Delta T=E_{EOM-IP-CCSDT-3/aug-cc-pVTZ}-E_{EOM-IP-CCSDT/aug-cc-pVTZ}.$$

These approaches are labelled EOM-IP-X/TQcCT and EOM-IP-X/CcCT, where X is CC3 or CCSDT-3. The EOM-IP family of approaches are computed using CFOUR [72]. These QFFs use the aug-cc-pCVTZ basis set [73] rather than the Martin–Taylor basis set used for the other QFFs as a matter of convenience for working with CFOUR.

The effect of additive corrections to the EOM-IP based QFFs are examined by providing spectroscopic data from QFFs which use only some components that make up the total EOM-IP-CCSDT-3/CcCT and EOM-IP-CCSDT-3/TQcCT QFFs. These include QFFs using a quadruple-zeta and quintuple-zeta Dunning basis set with no further corrections (EOM-IP-CCSDT-3/QZ and EOM-IP-CCSDT-3/5Z), QFFs using a three-point extrapolation (EOM-IP-CCSDT-3/TQ5) and a three-point extrapolation with a core correlation correction (EOM-IP-CCSDT-3/TQ5+cC). The additive corrections here are chosen partly for parity with other, established QFFs. Further corrections such as quantum electrodynamics effects may ultimately be necessary for spectroscopic accuracy [74,75], however, such treatment is beyond the scope of the present work.

Adiabatic excitation energies (AEEs) for the electronically excited state QFFs are provided by taking the difference of the excited state energy and the corresponding ground state energy (e.g., (T)+EOM/CcCR for the excited state and CcCR for the ground state) while also including the obtained anharmonic zero-point corrections and refitting energies from the QFFs.

EOM-IP based QFFs are computed for ground electronic states of the cationic species as well as the electronically excited states. High-resolution experimental data are available for these ground electronic states and can be used to validate the EOM-IP based QFFs. This ensures that the method’s predictions for hitherto unassigned frequencies of the higher states will be reliable. Additionally, ground state type CcCR and F12-TZ QFFs are undertaken for the $\tilde{A}$ ${}^{2}$A${}_{1}$ H${}_{2}$O${}^{+}$ state with the goal of providing highly accurate spectroscopic predictions from these reliable QFFs. This is feasible here because the drop in symmetry to C${}_{s}$ still results in a different electronic symmetry label for the $\tilde{X}$ and $\tilde{A}$ states. The caveat is that such results in uneven treatment of the Renner–Teller pair of this species. While this is not the case for the other species, computation of the available states in this way will allow for internal benchmarking of the pure electronically excited state QFFs when available.

## 3. Results

### 3.1. CO+

The results for all three states of CO${}^{+}$ studied herein—the $\tilde{X}$ ${}^{2}\Sigma^{+}$, $\tilde{A}$ ${}^{2}\Pi$ and $\tilde{B}$ ${}^{2}\Sigma^{+}$ states—are given in Table 1, Table 2 and Table 3, respectively. In addition to its role as a marker of cometary phenomena, CO${}^{+}$ is a useful test case for this group of open-shell cations and helps to understand the behavior of the electronically excited state QFFs investigated in the present work. Thus, this data will clarify which set of QFF data is reliable for spectroscopic prediction.

Anharmonic vibrational fundamental frequencies from all QFFs match well with experimental values for the $\tilde{X}$ state. No QFF here deviates more than 14 cm${}^{-1}$ from the literature values [17,18] for the C–O stretch fundamental frequency. Notably, the F12-TZ QFF matches with experiment to less than 1.0 cm${}^{-1}$. Rotational constants also compare reasonably with experimental values, with the closest QFF being CcCR, which places the B${}_{0}$ constant at 59,148 MHz compared to the experimental value of 59,270.5 MHz. EOM-IP-CCSDT/CcC, the highest-level EOM-IP based QFF employed here, differs from experiment by 16.8 cm${}^{-1}$ for $\nu_{1}$ and 149.5 MHz for B${}_{0}$. The other EOM-IP based approaches are somewhat closer, although this is likely fortuitous and overall they behave similarly to EOM-IP-CCSDT/CcC. All QFFs agree with the experimental value for the D${}_{e}$ distortion constant to within 3.0 kHz. Thus, both reference ground-state type QFFs and the EOM-IP family of QFFs, which may be conveniently applied to this ground electronic state for benchmarking, behave reasonably for this system.

The predictions for the H${}_{e}$ constant here may also be useful for fully fleshing out line list models for this system. CcCR places this constant at 122.642 mHz, whereas EOM-IP-CCSDT/CcC places it at 133.314 mHz. All other QFFs employed herein fall between these two values. The predictions for the B${}_{1}$ constant here may also be valuable owing to the high level of theory employed.

**Figure 1:**
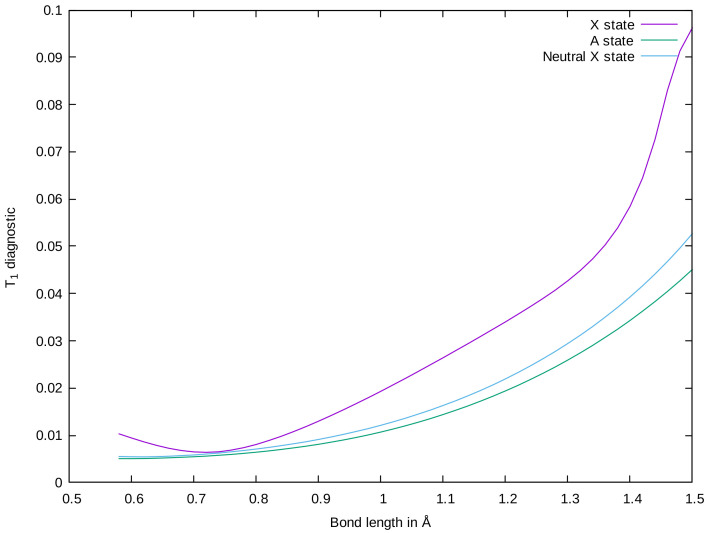
T${}_{1}$ Diagnostic vs. C–O bond length for selected electronic states of CO${}^{+}$ and neutral CO.

QFF performance for the electronically excited states of CO${}^{+}$ is not so straightforward. The (T)+EOM/CcCR method seems to have significant trouble in treating the $\tilde{A}$ ${}^{2}\Pi$ state of CO${}^{+}$ (Table 2). The fundamental vibrational frequency is nearly 100 cm${}^{-1}$ lower than in experimental data. Investigation of the T${}_{1}$ diagnostic [76] shows an abnormally high value of 0.035 near the equilibrium geometry of the $\tilde{A}$ ${}^{2}\Pi$ state (approximately 1.25 Å). Figure 1 depicts the T${}_{1}$ diagnostic of variationally accessible states of CO+ and of neutral CO. This high T${}_{1}$ diagnostic indicates that the $\tilde{X}$ ${}^{2}\Sigma^{+}$ state has significant multi-reference character at higher C–O bond lengths, and, thus, lays a poor foundation for (T)+EOM/CcCR’s treatment of the higher electronic states. By contrast, the ground electronic state of neutral CO is much better described by a single reference determinant at the equilibrium geometries of the $\tilde{A}$ ${}^{2}\Pi$ state. EOM-EE-CC3 exhibits similar issues as (T)+EOM/CcCR here and has a similar magnitude of error as (T)+EOM/CcCR compared to experiment for the $\nu_{1}$ frequency. For both of these QFFs, the H${}_{e}$ constant, with large negative values on the order of −400 mHz, disagrees significantly with the other QFFs, which place this constant around 30 mHz.

On the other hand, the EOM-IP approaches, which access the $\tilde{A}$ state by way of the closed-shell neutral’s ground electronic state, behave quite well in comparison to known values, with the high level EOM-IP-CCSDT/CcC QFF placing the result about 22.8 cm${}^{-1}$ higher than experimental data, as seen in Table 2. Most of the QFFs overestimate the experimental frequency to a similar magnitude, but this may be due to a weakness in treating diatomic species with the VPT2 approach used. The problem may also arise from the higher order C–O bond, which is somewhat problematic for QFFs [52,77,78]. This effect is likely exacerbated by the diatomic system, which has no counterbalancing contributions from other, well-behaved types of bonds. The B${}_{0}$ constant demonstrates similar behavior across QFFs as the fundamental vibrational frequency: significant divergence from experiment for (T)+EOM/CcCR and EOM-EE-CC3 based QFFs are in stark contrast to the reasonable agreement seen for EOM-IP based QFFs. The D${}_{e}$ and H${}_{e}$ constants for the former two are also significantly different than for the rest of the QFFs.

However, EOM-IP does not perform as well for the $\tilde{B}$ ${}^{2}\Sigma^{+}$ state. EOM-IP QFFs all overestimate this frequency by more than 100 cm${}^{-1}$. This may indicate potential pitfalls for EOM-IP based QFFs, possibly due to lower quality dynamic correlation in this instance. EOM-EE-CC3 based QFFs, however, perform quite well for this state.

The shorter equilibrium C-O bond length for the $\tilde{B}$ ${}^{2}\Sigma^{+}$ state results in a lower T${}_{1}$ diagnostic for the corresponding reference state and, hence, lower multi-reference character. This results in better behavior for the EOM-EE based treatments, as the EOM-EE energies are dependent on accurate ground-state coupled cluster amplitudes for the reference state. Despite this, (T)+EOM still exhibits pathological failure here. The excited electronic states of CO${}^{+}$, thus, offer an important lesson: an intelligent choice of method is likely necessary for studying properties of electronically excited states, and the application of QFFs to electronically excited states will likely depend on having a varied toolbox of methods. The right tool should be applied judiciously for a given system.

Comparison between TQcC QFFs and CcC QFFs is also necessary as the latter group of approaches is likely infeasible for larger systems. The two point T-Q extrapolation does not appear to harm performance much for these species. However, T-Q extrapolation without the quintuple-zeta contribution is less computationally demanding by a fair margin: walltimes for the $\tilde{A}$ ${}^{2}\Pi$ state for EOM-IP-CCSDT are 105 h at the CcC level compared to 22 h at the TQcC level. This reduction of more than 75% produces a difference of less than 1.0 cm${}^{-1}$ in the fundamental frequency between the two methods. In addition, there is minimal difference between EOM-IP-CC3 and EOM-IP-CCSDT-3 QFFs for CO${}^{+}$: 1555.3 cm${}^{-1}$ for the former vs 1554.9 cm${}^{-1}$ for the latter for the fundamental C–O stretch frequency. EOM-IP-CC3 is somewhat less expensive due to the inclusion of less triples terms. Both approximate the highly expensive EOM-IP-CCSDT QFF reasonably well with a difference of less than 2.0 cm${}^{-1}$ for the CcC variants.

Table 1, Table 2 and Table 3 also provide spectroscopic data from QFFs which use only some pieces of the overall composite EOM-IP-CCSDT-3/CcCT and EOM-IP-CCSDT-3/TQcCT QFFs. First examining basis set size, the $\nu_{1}$ fundamental frequency for $\tilde{X}$ ${}^{2}\Sigma^{+}$ CO${}^{+}$ changes from 2151.0 cm${}^{-1}$ for a QZ basis set to 2155.6 cm${}^{-1}$ for a 5Z basis set. A three-point T-Q-5 extrapolation continues this trend, with a value of 2160.3 cm${}^{-1}$. This hierarchical convergence is also seen for the $\tilde{A}$ ${}^{2}\Pi$ state. The $\tilde{B}$ ${}^{2}\Sigma^{+}$ state shows a similar trend going from QZ to 5Z, however the T-Q-5 extrapolation lowers the anharmonicity to 1798.9 cm${}^{-1}$ from the 5Z value of 1793.3 cm${}^{-1}$. The core-correlation correction contributes no more than 10 cm${}^{-1}$ difference to any of these frequencies. The higher-order correlation correction (“T”), results in a large increase of more than 30 cm${}^{-1}$ for $\nu_{1}$ for the $\tilde{X}$ ${}^{2}\Sigma^{+}$ state and results in closer agreement with experiment and ground state QFFs compared to not including this correction.

Based on experimental comparisons, CcCR and F12-TZ data are trustworthy for the $\tilde{X}$ ${}^{2}\Sigma^{+}$ and $\tilde{A}$ ${}^{2}\Pi$ states. For the $\tilde{B}$ ${}^{2}\Sigma^{+}$ state, EOM-EE-CC3 QFFs seem well-behaved although they fail catastrophically for the $\tilde{A}$ ${}^{2}\Pi$ state. The present CO${}^{+}$ data do not clearly favor EOM-IP or EOM-EE based QFFs for future application to electronically excited states which are not variationally accessible and suggest that intelligent method application is important for complex open shell cationic species.

Finally, novel high-level data is provided for some spectroscopic constants of these electronic states of CO${}^{+}$ in addition to the benchmarking outlined above: H${}_{e}$ is predicted for each for each electronic state, which was not available from the existing high resolution electronic studies. Across all non-pathological levels of theory, the H${}_{e}$ constant differs substantially across the three electronic states present herein: EOM-IP-CCSDT/CcC places it at 133.314 mHz, 8.936 mHz, and −0.002 mHz for the $\tilde{X}$ ${}^{2}\Sigma^{+}$, $\tilde{A}$ ${}^{2}\Pi$, and $\tilde{B}$ ${}^{2}\Sigma^{+}$ states, respectively. Thus, models utilizing this constant may provide additional clarity for identification of CO${}^{+}$ spectral signatures. B${}_{1}$ is also provided herein. Referring to EOM-IP-CCSDT/CcC values as qualitatively representative, B${}_{1}$ is 58558 MHz for the $\tilde{X}$ ${}^{2}\Sigma^{+}$ state. The $\tilde{B}$ ${}^{2}\Sigma^{+}$ state value is 53604 MHz, while there is a more significant shift for the $\tilde{A}$ ${}^{2}\Pi$ state at 46983.5 MHz. Although data from previous theory are available [15] since CO+ is a simple diatomic, the high level anharmonic treatment from established QFF approaches provided here should be useful in fully modeling CO${}^{+}$ spectra.

### 3.2. H${}_{2}$O${}^{+}$

#### 3.2.1. $\tilde{X}$ ${}^{2}$B${}_{1}$ H${}_{2}$O${}^{+}$

Anharmonic vibrational frequencies, provided in Table 4 for F12-TZ, CcCR, and the family of EOM-IP QFFs, agree excellently with the given experimental data for the $\tilde{X}$ ${}^{2}$B${}_{1}$ ground state of H${}_{2}$O${}^{+}$. Of these, the CcCR QFF is expected to perform the best due to the high-quality basis set extrapolation used as well as the additional corrective terms included. In addition, CcCR being a variational ground state method results in better treatment of dynamic correlation. CcCR places the $\nu_{1}$ frequency at 3260.2 cm${}^{-1}$, which agrees to within 1.2 cm${}^{-1}$ of 3259.04 cm${}^{-1}$ from gas phase experiments of Dinelli et al. and Huet et al. [79,80]. CcCR also agrees well the experimental value of 3212.86 cm${}^{-1}$ for $\nu_{2}$ given in several studies [20,79,80,81]. Literature values for the $\nu_{3}$ frequency is reasonably unanimous in assignment at 1408.42 cm${}^{-1}$ [20,36,37]. CcCR predicts this frequency to be 1408.3 cm${}^{-1}$. Agreement is generally quite close between CcCR and F12-TZ, as is often the case, with a mean absolute difference (MAD) of 2.6 cm${}^{-1}$ for F12-TZ vs. CcCR for the fundamental vibrational frequencies.

Notably, EOM-IP-CCSDT/CcC achieves quite reasonable agreement with experiment as well, with a mean absolute error (MAE) of 8.8 cm${}^{-1}$. Although this approach uses costly full triples, the treatment of dynamic correlation for EOM based energies tends to be worse. Hence, EOM-IP-CCSDT/CcC underperforming compared to CcCR is not surprising. This QFF could also potentially be improved by the inclusion of scalar relativistic corrections in future work. Similar to CO${}^{+}$, the EOM-IP-CCSDT-3 and EOM-IP-CC3 approaches approximate the EOM-IP-CCSDT method quite well with EOM-IP-CCSDT-3/CcC almost exactly matching the EOM-IP-CCSDT values. Vibrational fundamentals generated from both VCI and VPT2 calculations for the EOM-IP-CCSDT/CcC QFF agree within a few cm${}^{-1}$. Thus, the VPT2 calculations here are likely behaving appropriately. Breakdowns of the individual contributions to the EOM-IP-CCSDT-3 QFFs are also given in Table 4, which do not show any anomalous behavior and the overall effect of each of the corrections appears to be small. Increasing basis set quality from QZ, 5Z, to a three-point extrapolation appears to result in systematic convergence of frequencies, as is to be expected.

The CcCR harmonic frequencies also agree to within 6.0 cm${}^{-1}$ of the previous theoretical work by Feller and Davidson [38], which uses a similar CCSD(T) approach with additive corrections. EOM-IP-CCSDT/CcC, the most high-level EOM-IP based QFF employed here, corroborates with these harmonic frequencies reasonably well with no more than an 11.0 cm${}^{-1}$ deviation from the CcCR values. The less expensive EOM-IP approaches differ by no more than a few additional cm${}^{-1}$. F12-TZ reproduces CcCR fairly well. It agrees to within 5.0 cm${}^{-1}$ for all three fundamental frequencies. The anharmonic assignments given here for the ground state of H${}_{2}$O${}^{+}$ are therefore reliable.

The rotational constants for the $\tilde{X}$ ${}^{2}$B${}_{1}$ state of H${}_{2}$O${}^{+}$ are given in Table 5. CcCR has an MAE of 3415 MHz, or 0.4% error with the assignments from Muller et al. [83]. F12-TZ presents an MAE of 2812 MHz, surprisingly closer to experiment. The core correlation and relativistic effects included in CcCR are often necessary for accurate description of rotational constants [77]. Consequently, F12-TZ being more successful for the ground state of H${}_{2}$O${}^{+}$ is unusual, which may indicate some minor issues with CcCR here.

EOM-IP-CCSDT/CcC has an MAE of 3807 MHz for rotational constants compared to values from Muller et al., which is a respectable performance compared to CcCR at only a 392 MHz difference. Furthermore and once more, there is marginal difference between the very costly EOM-IP-CCSDT/CcC and cheaper approximations. EOM-IP-CCSDT-3/CcCT is actually marginally closer to experiment with an MAE 3755 MHz. There is also a marginal difference here between EOM-IP-CCSDT-3/TQcCT and EOM-IP-CCSDT-3/CcT: an MAD of 643 MHz between the two. Likewise, EOM-IP-CC3/CcCT and EOM-IP-CCSDT-3/CcCT have an MAD of only 725 MHz. Lastly, variations between rotational constants at different levels of theory are most severe in the A rotational constants [77]; EOM-IP-CCSDT-3/CcCT has an MAE of only 643 MHz for the B and C rotational constants compared to experiment. Distortion constants and geometrical parameters for this state are given in Table A1 in Appendix B.

Overall, the ground state QFFs describe the $\tilde{X}$ ${}^{2}B_{1}$ state of H${}_{2}$O${}^{+}$ quite well, as does the family of EOM-IP QFFs. The latter’s reasonable performance here, where high quality experimental data is available, is encouraging for its further application to systems and states herein with missing data.

#### 3.2.2. $\tilde{A}$ ${}^{2}$A${}_{1}$ H${}_{2}$O${}^{+}$

Table 6 gives harmonic and anharmonic vibrational frequencies for the $\tilde{A}$ ${}^{2}$A${}_{1}$ state of H${}_{2}$O${}^{+}$. The variational F12-TZ and CcCR approaches agree moderately well with results of Truong et al. [25] in consideration of the wide range of error reported for experiment. CcCR places the $\nu_{1}$ symmetric stretch at 3232.1 cm${}^{-1}$ compared to the experimental value of 3153 ± 169 cm${}^{-1}$. The CcCR value for the $\nu_{2}$ bending frequency, at 995 cm${}^{-1}$, is slightly outside of the reported margin of error at 903 ± 80 cm${}^{-1}$. A similar case is seen for the $\nu_{3}$ anti-symmetric stretch, placed at 3420.7 cm${}^{-1}$ for CcCR compared to 3331 ± 24 cm${}^{-1}$ from Truong et al. The value from Reutt et al. [20] for the $\nu_{1}$ frequency of 3547 ± 16 cm${}^{-1}$ disagrees both with the values of Truong et al. and theory presented herein. CcCR and F12-TZ agree to within 5.0 cm${}^{-1}$ for $\nu_{1}$ and $\nu_{2}$, with a difference of 41.0 cm${}^{-1}$ for the $\nu_{3}$ bending motion, with F12-TZ predicting the higher frequency. These methods also compare well with the harmonic frequencies provided by previous authors [38], validating the approach in the present study.

Special mention should be given to these methods’ uneven treatment of the Renner–Teller pair: uneven occupation of two π orbitals, which are degenerate at linearity, in each of these methods leads to a breaking of said degeneracy. EOM-EE-CC3 QFFs are not investigated for the $\tilde{A}$ ${}^{2}$A${}_{1}$ state for this reason. However, based on moderately close experimental comparisons given above, the uneven treatment of these orbitals does not seem to have catastrophic consequences. This is probably because the deviation from degeneracy is small.

Moving on to the AEEs (i.e., the barrier to linearity from the $\tilde{X}$ ${}^{2}$B${}_{1}$ state to the $\tilde{A}$ ${}^{2}$A${}_{1}$ state), the MRCI work of Brommer et al. [35] calculates a value of 7886 cm${}^{-1}$, which agrees reasonably with the values given in Table 6 for most levels of theory, except for EOM-IP-CC3. The closest value is 7950.1 cm${}^{-1}$ from CcCR, highlighting the utility of these variationally-accessible QFFs in providing reliable estimates of missing spectroscopic data.

The work herein overall supports the qualitative positions of the band origins of this state given by Truong et al. [25], with potential guidance as to the more exact positions of the band origins. The full set of rotational constants, distortion constants, and geometrical parameters predicted by these and other levels of theory herein are given in Table A2 for the $\tilde{A}$ ${}^{2}$A${}_{1}$ state. As these are not widely available at high levels of theory [2], these data could prove useful in modeling complete UV/Vis spectra of H${}_{2}$O${}^{+}$.

Turning to analysis of the pure electronically excited state QFFs, EOM-IP-CCSDT/CcC matches within reported margins of error for experimental frequencies from Truong et al. [25] for the symmetric stretch as well as the bending motion of $\tilde{A}$ ${}^{2}$A${}_{1}$ H${}_{2}$O${}^{+}$: $\nu_{1}$ (the symmetric stretch) is 3222.0 cm${}^{-1}$ at the EOM-IP-CCSDT/CcC level compared to the experimental value of 3153 ± 169 cm${}^{-1}$, while $\nu_{2}$ (the bend) is placed at 970.2 cm${}^{-1}$ compared to 903 ± 80 cm${}^{-1}$ from experiment. Placement of the $\nu_{3}$ anti-symmetric stretch is somewhat higher at 3405.3 cm${}^{-1}$ compared to Truong et al.’s value of 3331 ± 24 cm${}^{-1}$. However, EOM-IP-CCSDT/CcC still has the best performance of all the QFFs employed for comparison to known data, even better than the variationally accessible F12-TZ and CcCR approaches. Application of EOM-IP-CCSDT/CcC to the $\tilde{B}$ ${}^{2}$B${}_{1}$ state, which is not variationally accessible, should therefore be trustworthy. Replacing the costly EOM-IP-CCSDT method with CCSDT-3 and CC3 variants seems to harm the description of the $\nu_{2}$ bending frequency significantly, unlike in other systems where the two are well behaved. EOM-IP-CCSDT-3/CcCT places the $\nu_{2}$ frequency higher at 1067.1 cm${}^{-1}$, while EOM-IP-CC3/CcCT places it significantly lower at 690.4 cm${}^{-1}$. Upon closer examination, the higher placement from EOM-IP-CCSDT-3/CcCT appears to result from the “T” higher-order correlation correction, suggesting that this correction may have issues with the highly anharmonic bending motion. Basis set convergence is also not straightforward for this motion, with EOM-IP-CCSDT-3/QZ placing the motion at 906.9 cm${}^{-1}$, EOM-IP-CCSDT-3/5Z placing it at 900.5 cm${}^{-1}$ and EOM-IP-CCSDT-3/TQ5 placing it at 912.9 cm${}^{-1}$.

(T)+EOM/CcCR and variationally-accessible ground state-style CcCR QFFs show reasonably close agreement in the vibrational frequencies for the $\tilde{A}$ ${}^{2}$A${}_{1}$ state of H${}_{2}$O${}^{+}$, given in Table 6. There is only a 0.4 cm${}^{-1}$ difference between the two for the $\nu_{2}$ frequency, and a difference of 4.5 cm${}^{-1}$ for the $\nu_{1}$ frequency. The discrepancy in the $\nu_{3}$ frequency is more significant, with (T)+EOM/CcCR placing this nearly 60 cm${}^{-1}$ higher in frequency at 1056.6 cm${}^{-1}$. (T)+EOM/CcCR thus seems well behaved for this state, in contrast to its performance for the higher electronic states of CO${}^{+}$.

EOM-IP-CCSDT/CcC has an MAD of 911.18 MHz compared to CcCR for rotational constants (Table A2). Again, this deviation is possibly due to the latter’s uneven Renner–Teller treatment. Reasonable agreement is shown between (T)+EOM/CcCR and CcCR with an MAD of 564.2 MHz or 0.2% error. Overall and again, the data for this state shows that EOM-IP-CCSDT/CcC seems to be a valid approach for treating this state, as EOM-IP provides a balanced treatment of the Renner–Teller pair. However, this approach is very costly and not easy to extrapolate to larger systems. Hence, the large difference between CCSDT-3 and CC3 variants in their description of the bending motion is disappointing. This issue may be due to the highly anharmonic nature of this mode, however, and the perturbative triples variants may perform better with more well behaved states.

The $\nu_{2}$ bending motion of this state is worth some further consideration. This motion is highly anharmonic with a positive anharmonicity of more than 500 cm${}^{-1}$ at the EOM-IP-CCSDT/CcC level with VPT2 (424.1 cm${}^{-1}$ for $\omega_{2}$ compared to 970.2 cm${}^{-1}$ for $\nu_{2}$).

VCI calculations give an anharmonic frequency of 557.535 cm${}^{-1}$ at the EOM-IP-CCSDT/CcC, markedly different from the VPT2 result of 970.2 cm${}^{-1}$ for the $\nu_{2}$ bend. Thus, although VPT2 and VCI results agree closely for the other two modes, a VPT2 based treatment of the bend for the $\tilde{A}$ ${}^{2}A_{1}$ state of H${}_{2}$O${}^{+}$ is likely inadequate. The highly anharmonic nature of this mode, combined with difficulties in properly handling the Renner–Teller effect’s breakdown of the Born–Oppenheimer approximation, means that semi-global potential surfaces with multi-reference methods may be necessary for confident characterization of this state. There is considerable difficulty in determining experimental fundamental frequencies for the bending motion due to the vibrational progression and low intensity of the fundamental and lower overtones of this mode [24,35]. Truong et al. give fairly large margins of error at as much as 169 cm${}^{-1}$, and values from the previous work by Reutt et al. [20] differ by as much as 400 cm${}^{-1}$ from Truong et al. for the symmetric H–O stretch frequency. Thus, ambiguity in proper assignment of this mode remains, which could possibly be clarified with a more thorough theoretical treatment with semi-global potential surfaces.

#### 3.2.3. $\tilde{B}$ ${}^{2}$B${}_{2}$ H${}_{2}$O${}^{+}$

Large discrepancies exist between previous experimental assignments and the theoretical results given here for the $\tilde{B}$ ${}^{2}$B${}_{2}$ state of H${}_{2}$O${}^{+}$ in Table 7. However, Feller and Davidson [38] point out that the assignments given by Truong et al. [25] for this state likely result from misassignment of the origin band, which is supported by the discrepancy between all of the QFFs presented here as well as other experimental data both for the fundamental frequencies and the AEE. The AEE given by Reutt et al. [20] of 36,757 cm${}^{-1}$ is about 3000 cm${}^{-1}$ higher than that of all QFFs, a difference of approximately 8%. This lends credence to the assertions of Feller and Davidson of possible misassignment of the origin band. Harmonic frequencies at the CCSD(T)/aug-cc-pV5Z level with additional corrections calculated by Feller and Davidson [38] agree quite closely with those of (T)+EOM/CcCR QFF as well as those for the EOM-EE-CC3/TQcC QFF. The EOM-IP family places these harmonic frequencies relatively higher, e.g. $\omega_{3}$ is 1985.1 cm${}^{-1}$ for EOM-IP-CCSDT/CcC compared to 1945.1 cm${}^{-1}$ from Feller and Davidson. Frequencies from the other EOM-IP based QFFs closely approximate the EOM-IP-CCSDT/CcC values. Examining the effect of the individual components of the composite EOM-IP QFFs shows that the “T” correction has the largest effect here for the EOM-IP-CCSDT-3 QFFs, lowering the $\omega_{2}$ harmonic frequency for the bend by more than 10 cm${}^{-1}$ and bringing the value more closely in line with the previous work of Feller and Davidson.

As experimental comparisons are problematic, estimating which set of data and, hence, which method likely produces the most meaningful novel constants is more accurate is difficult. Also, issues with the present adiabatic approach may exist due to the complex potential surface near the well of the $\tilde{B}$ ${}^{2}$B${}_{2}$ state [27]. VCI calculations at the EOM-IP-CCSDT/CcC level show some noteworthy discrepancies. The $\nu_{1}$ symmetric stretch differs 23.8 cm${}^{-1}$ between VPT2 and VCI for the EOM-IP-CCSDT/CcC QFF, which appears to be due to differences in Fermi resonances with the 2$\nu_{2}$ and 2$\nu_{3}$ overtone bands. The $\nu_{2}$ bending motion differs more severely, at 1478.9 cm${}^{-1}$ for VPT2 compared to 1561.7 cm${}^{-1}$ for VCI, a difference of 83 cm${}^{-1}$. This suggest unusual anharmonicity for this frequency. These VCI results thus suggest some caution in interpreting the VPT2 QFFs for this state. Nevertheless, the present work provides an alternative to a costly and complex diabatic treatment and may be useful in guiding interpretation of future experimental work. Based on adequate performance for CO${}^{+}$ and the other electronic states of H${}_{2}$O${}^{+}$, the EOM-IP-CCSDT/CcC QFF computed for this state likely offers reasonable estimations of spectroscopic data from the present group of adiabatic approaches.

All QFF approaches, as well as previous theory [38], report a significant lowering of the symmetric and antisymmetric stretching frequencies for the $\tilde{B}$ ${}^{2}B_{2}$ state of H${}_{2}$O${}^{+}$ relative to the values of the $\tilde{X}$ ${}^{2}B_{1}$ state. For example, EOM-IP-CCSDT/CcC places the $\nu_{1}$ and $\nu_{3}$ frequencies at 2409.3 cm${}^{-1}$ and 1785.7 cm${}^{-1}$, respectively, for the $\tilde{B}$ ${}^{2}B_{2}$ state, compared to 3203.0 cm${}^{-1}$ and 3248.7 cm${}^{-1}$ for the $\tilde{X}$ ${}^{2}B_{1}$ state. The geometrical parameters for this state, given in Table A4, showcase an increase in the r${}_{0}$(H-O) bond length of 0.125 Å. This is a reasonable explanation for the dramatic decrease in frequency of these motions when combined with the dramatically smaller H-O-H bond angle of 57.662 ${}^{\circ }$ and the change in electronic configuration—singly occupying the out-of-plane b${}_{1}$ π orbital rather than the b${}_{2}$ π orbital as in the ground state. The shift in frequency for the bending motion, $\nu_{2}$, is not as severe but is still marked: 1597.1 cm${}^{-1}$ for the $\tilde{B}$ ${}^{2}B_{2}$ state compared to 1402.3 cm${}^{-1}$ for the ground state. These large shifts should provide a clear disambiguation for the presence of this electronic state of H${}_{2}$O${}^{+}$ in cometary spectra. Rotational constants for all QFFs for the $\tilde{B}$ ${}^{2}B_{2}$ state of H${}_{2}$O${}^{+}$ are given in Table A3, while geometrical parameters and distortion constants are given in Table A4 and should prove useful in modeling spectral features.

Lastly, EOM-IP-CCSDT-3/CcCT seems to act as a reasonable, cost-effective approximation to EOM-IP-CCSDT/CcC here. Anharmonic fundamental vibrational frequencies differ no more than 4 cm${}^{-1}$. EOM-IP-CCSDT/CcC ran with a walltime of 1253 h compared to 108.5 h for EOM-IP-CCSDT-3/CcCT. As a result, the latter may be a prudent choice for application to larger systems where prohibitive scaling will make the former infeasible. EOM-IP-CCSDT-3/TQcCT may also be a viable choice for a further shaving-off of computational cost, as its frequencies differ by no more than 7 cm${}^{-1}$ from EOM-IP-CCSDT/CcC.

### 3.3. H${}_{2}$CO${}^{+}$

#### 3.3.1. $\tilde{X}$ ${}^{2}$B${}_{2}$ H${}_{2}$CO${}^{+}$

Reasonable agreement is exhibited between the CcCR and F12-TZ QFFs and available experimental frequencies (Table 8) for the $\tilde{X}$ ${}^{2}$B${}_{2}$ state of H${}_{2}$CO${}^{+}$. EOM-IP-CCSDT-3/TQcCT actually outperforms both ground-state QFFs in comparison to several experimental frequencies: $\nu_{4}$ is computed to have a value of 1039.2 cm${}^{-1}$ at the EOM-IP-CCSDT-3/TQcCT level, compared to the experimental value of 1036 ± 4 cm${}^{-1}$. F12-TZ places this frequency at 1013.6 cm${}^{-1}$ while CcCR places it at 1007.9 cm${}^{-1}$. EOM-IP-CCSDT-3/TQcCT also outperforms the ground state methods for the $\nu_{5}$ anti-symmetric C–H stretch. Additionally, VCI calculations agree within several cm${}^{-1}$ of the VPT2 results for EOM-IP-CCSDT-3/TQcCT, suggesting the VPT2 QFFs are well behaved for this state. The core correlation correction here makes a large difference in the $\nu_{2}$ O–C stretching frequency, increasing it by about 6.6 cm${}^{-1}$. The “T” correction has, again, the largest effect overall and brings many of the fundamental frequencies in closer agreement with experiment, with the only exception being the $\nu_{1}$ symmetric C-H stretch. EOM-IP-CCSDT-3/TQcCT is the only EOM-IP based QFF employed here, owing to its adequate performance for smaller test cases as well as time and cost constraints.

CcCR places the $\nu_{6}$ frequency nearly 60 cm${}^{-1}$ lower than the experimental value. F12-TZ places this much closer to the experimental value (823.7 cm${}^{-1}$) at 825.0 cm${}^{-1}$. This may be due to known issues with the CcCR approach at handling low-frequency, large amplitude motions [86]. Overall, comparison with experimental data for this state suggests that the EOM-IP-CCSDT-3/TQcCT QFF likely provides a valid prediction of spectroscopic constants for the electronically excited states. Rotational constants and distortion constants are given in Table A5 and Table A6, respectively, where there is an MAD of 136 MHz between EOM-IP-CCSDT-3/TQcCT and CcCR in the rotational constants. The vibrationally-corrected rotational constants provided herein are not available experimentally, and the high-level CcCR computations given here are, thus, likely the most reliable estimates produced for these constants.

#### 3.3.2. $\tilde{A}$ ${}^{2}$B${}_{1}$ H${}_{2}$CO${}^{+}$

Frequencies for the $\tilde{A}$ ${}^{2}$B${}_{1}$ state of H${}_{2}$CO${}^{+}$ are given in Table 9. Experimental frequencies are available for the O–C stretch and the H–C–O symmetric bending motions, given at 1488 cm${}^{-1}$ [29,30] and 1250 cm${}^{-1}$ [28,29,30], respectively. EOM-IP-CCSDT-3/TQcCT matches these frequencies well: predicting the O–C stretch to be 1489.3 cm${}^{-1}$, only 1.3 cm${}^{-1}$ higher than the literature value and well within the reported margin of error. The H–C–O symmetric bend is computed to be 1262 cm${}^{-1}$ by this method, only a bit higher than the literature value of 1250 cm${}^{-1}$. The AEE computed by EOM-IP-CCSDT-3/TQcCT is 26,469.6 cm${}^{-1}$, 540.6 cm${}^{-1}$ higher than the literature value of 25929 ± 5 cm${}^{-1}$ [28,29,30], a relatively small error compared to CO${}^{+}$ and H${}_{2}$O${}^{+}$.

VCI and VPT2 results for EOM-IP-CCSDT-3/TQcCT are generally in close agreement, with the largest difference being the $\nu_{2}$ frequency. VCI places this frequency at 1505.7 cm${}^{-1}$, 16.4 cm${}^{-1}$ higher than the VPT2 value of 1489.3 cm${}^{-1}$. The “T” correction here brings both the $\nu_{2}$ and $\nu_{3}$ frequencies much closer to the experimental values. The core-correlation correction also has a large effect on several modes, notably the $\nu_{3}$ H–C–O symmetric bend, which drops by nearly 34 cm${}^{-1}$ when comparing EOM-IP-CCSDT-3/TQ to EOM-IP-CCSDT-3/TQ+cC. Several other motions exhibit large changes due to the core correlation correction, and it is likely important here due to the O–C double bond.

(T)+EOM/CcCR performs reasonably for the $\nu_{2}$ O–C stretch at 1470.1 cm${}^{-1}$. However, its H–C–O symmetric bend is 1226.4 cm${}^{-1}$ or 23.6 cm${}^{-1}$ lower than in experiment. The T${}_{1}$ diagnostic at the CCSD/aug-cc-pV5Z level for the equilibrium geometry of the $\tilde{A}$ ${}^{2}$B${}_{1}$ state is relatively high at 0.037. This suggests that (T)+EOM/CcCR may have issues here due to the reference state’s high multi-reference character, as was seen with CO${}^{+}$.

Experimental frequencies are unavailable for the other vibrational motions. The novel data predicted by EOM-IP-CCSDT-3/TQcCT, which seems well behaved for H${}_{2}$CO${}^{+}$, should, therefore, make valuable predictions for these band origins. Several vibrational motions differ significantly from the $\tilde{X}$ ${}^{2}B_{1}$ state: the $\nu_{1}$, $\nu_{2}$, $\nu_{4}$, and $\nu_{5}$ frequencies all differ by more than 150 cm${}^{-1}$ between electronic states. The $\tilde{A}$ ${}^{2}B_{1}$ state is formed by a π→n transition from the doubly occupied $b_{1}$ orbital into the singly occupied $b_{2}$ orbital resulting in a lengthening of the C–O bond to 1.338 Å from 1.193 Å from the ground electronic state. This explains the drop in the O–C stretching frequency from 1677.3 cm${}^{-1}$ to 1489.3 cm${}^{-1}$. As the other geometrical parameters do not change much, the shift in electronic density to the non-bonding orbital, which is primarily centered on the oxygen, is likely responsible for the changes in the other frequencies.

Rotational constants, distortion constants and geometrical parameters for this state predicted herein are given in Table A7 and Table A8. The EOM-IP-CCSDT-3/TQcCT values for these parameters will be useful in producing full vibronic spectra of H${}_{2}$CO${}^{+}$ and enhance understanding of its potential role in cometary phenomena. The A${}_{0}$ constant increases by 1760 MHz from the $\tilde{X}$ ${}^{2}B_{1}$ state value of 267989 MHz while the B${}_{0}$ changes more significantly, decreasing by 7444 MHz from the ground state value. Several distortion constants also change to an appreciable degree and should help in matching features based on qualitative assessment of spectral signatures.

#### 3.3.3. $\tilde{B}$ ${}^{2}$A${}_{1}$ H${}_{2}$CO${}^{+}$

Table 10 contains the fundamental vibrational frequencies of the $\tilde{B}$ ${}^{2}A_{1}$ state of H${}_{2}$CO${}^{+}$. Experimental assignment is available for the $\nu_{2}$ frequency, which is placed at 1304 cm${}^{-1}$ [28,29,30]. There has been controversy with which totally symmetric mode this fundamental corresponds [87]. Although Niu et al. [29] assign this band to the $\nu_{2}$ C–O stretching motion, the present results support assignment of the experimental 1304 cm${}^{-1}$ band origin to the $\nu_{3}$ H–C–O symmetric bend, instead. EOM-IP-CCSDT-3/TQcCT places $\nu_{3}$ at 1326.4 cm${}^{-1}$ and $\nu_{2}$ at 1360.0 cm${}^{-1}$. Looking at the AEEs, EOM-IP-CCSDT-3/TQcCT is a mere 46.2 cm${}^{-1}$ (0.11%) off from the literature value of 39,928 cm${}^{-1}$. By contrast, (T)+EOM/CcCR is off by 955 cm${}^{-1}$, a much larger percent error of 2.4 % but still better than many of the other molecular states reported above in this work. Therefore, EOM-IP-CCSDT-3/TQcCT is likely the more trustworthy of the two methods employed here, in line with the results for the $\tilde{A}$ ${}^{2}B_{1}$ state of H${}_{2}$CO${}^{+}$.

VCI and VPT2 comparisons for this state at the EOM-IP-CCSDT-3/TQcCT level show some notable discrepancies. The frequencies for $\nu_{2}$ differ by 14 cm${}^{-1}$ while the $\nu_{3}$ frequencies differ by 20 cm${}^{-1}$. These differences may be explained by the presence of a Darling–Dennison type resonance between these two modes which are not present in the VPT2 calculations. $\nu_{1}$ shows a larger difference between the two approaches, with VPT2 placing the frequency at 2734.8 cm${}^{-1}$ compared to 2653.8 cm${}^{-1}$ for VCI. This difference appears to arise from differences in the Fermi resonance polyad of $\nu_{2}$ = $\nu_{3}$ + $\nu_{4}$ = 2$\nu_{3}$ = 2$\nu_{4}$, with the contributions to the VCI polyad being higher in energy. Looking at the breakdown of individual contributions to the EOM-IP-CCSDT-3/TQcCT composite QFF, the core correlation (“cC”) correction does not appear to have as significant a contribution here as with the $\tilde{A}$ ${}^{2}B_{1}$ state, although the contributions of the “T” correction appear to be of similar magnitude.

Since the EOM-IP-CCSDT-3/TQcCT results here seem trustworthy based on experimental comparison for the $\nu_{2}$ frequency and also taking into consideration its performance for the other states of H${}_{2}$CO${}^{+}$, the novel data for the missing fundamental frequencies and rotational constants should prove valuable for modeling the UV/Vis transitions of H${}_{2}$CO${}^{+}$ in cometary environments. The $\nu_{1}$ frequency for the $\tilde{B}$ ${}^{2}A_{1}$ state is closer to the $\tilde{X}$ ${}^{2}B_{1}$ state value at 2733.8 cm${}^{-1}$ compared to the shift seen for the $\tilde{A}$ ${}^{2}B_{1}$ state. The $\nu_{2}$ frequency at 1360.0 cm${}^{-1}$, however, is even lower than the $\tilde{A}$ ${}^{2}B_{1}$ state value. The r${}_{0}$(O-C) bond length at 1.285 Å is shorter than the value for the $\tilde{A}$ ${}^{2}B_{1}$ state (1.338 Å), marking this behavior as unexpected. The $\tilde{B}$ ${}^{2}A_{1}$ state is formed by a σ→n excitation, where the σ $a_{1}$ orbital is centered on the C–O bond, and the non-bonding $b_{2}$ orbital is centered on the oxygen. This weakening of the σ bond explains the larger decrease in vibrational frequency. Other frequencies, such as the $\nu_{5}$ anti-symmetric C-H stretch, are, like $\nu_{1}$, generally closer to the $\tilde{X}$ ${}^{2}B_{1}$ values.

The feature at 1304 cm${}^{-1}$, discussed above and attributed here to the H-C-O bend with a value of 1326.4 cm${}^{-1}$ at the EOM-IP-CCSDT-3/TQcCT level, is at least 50 cm${}^{-1}$ away from any band origins from other electronic states, and is likely a useful indicator of the $\tilde{B}$ ${}^{2}A_{1}$ in analyzing H${}_{2}$CO${}^{+}$ spectra. Rotational constants for the $\tilde{B}$ ${}^{2}A_{1}$ state are given in Table A9. Distortion constants and geometrical parameters are given in Table A10.

## 4. Conclusions

Several high level approaches were explored for evaluating the spectroscopic data of CO${}^{+}$, H${}_{2}$O${}^{+}$ and H${}_{2}$CO${}^{+}$. Newly employed EOM-IP based quartic force fields with higher-order correlation correction and core correlation perform well in comparison to available experimental benchmarks in many cases. EOM-IP-CCSDT-3/TQcCT outperforms ground-state QFFs in its treatment of several modes of the $\tilde{X}$ ${}^{2}B_{1}$ state of the formaldehyde cation. New data are provided using this QFF for missing fundamental vibrational frequencies of several states of H${}_{2}$CO${}^{+}$, as well as vibrationally-excited rotational constants and distortion constants for the electronically excited states of all molecules studied herein. Additionally, work done in the present study as well as by previous authors supports reassignment of the fundamental vibrational frequencies for the $\tilde{B}$ ${}^{2}B_{1}$ state of H${}_{2}$O${}^{+}$ to approximately 2409.3 cm${}^{-1}$, 1478.9 cm${}^{-1}$, and 1785.7 cm${}^{-1}$.

Many of the EOM-IP based approaches herein, especially the high-level EOM-IP-CCSDT/CcC approach, are infeasible for larger systems due to prohibitive computational cost. The EOM-IP-CCSDT-3/TQcCT QFF seems to be a promising avenue for treating small systems of astrochemical interest such as the small molecular cations in the present study. Even so, this method fails for the $\tilde{B}$ $\Sigma^{+}$ state of CO${}^{+}$. Additionally, VCI calculations highlight some issues with the VPT2 based treatment for several highly anharmonic motions of the electronically excited states of H${}_{2}$O${}^{+}$. These concerns highlight the need for a judicious choice of method when dealing with troublesome electronic states. The previously explored (T)+EOM/CcCR seems to behave poorly for the molecules in the present study, and its usage may be limited to more well-behaved systems. Pathological systems like the $\tilde{A}$ ${}^{2}A_{1}$ state of H${}_{2}$O${}^{+}$ or the higher electronic states of CO${}^{+}$ may necessitate semi-global potential surfaces for truly accurate treatment but the QFFs presented herein represent a reasonable attempt at making a simple, useful approximation. The new data provided by this paper will be of astrochemical interest for modeling molecular transitions with application to cometary phenomena. Examples of such data include the fundamental vibrational frequencies of the $\tilde{A}$ ${}^{2}B_{1}$ and $\tilde{B}$ ${}^{2}A_{1}$ states of H${}_{2}$CO${}^{+}$ (e.g., 1489.3 cm${}^{-1}$ and 1360.0 cm${}^{-1}$ for the O-C stretch of both states, respectively) and the tentative reassignments for $\tilde{B}$ ${}^{2}B_{1}$ H${}_{2}$O${}^{+}$. These new data are necessary for a complete spectral model of electronic excitations, and should be useful for high resolution vibrational studies using ground-based observatories and even the Hubble Space Telescope.

## Figures and Tables

**Table 1:** $\tilde{X}$${}^{2}\Sigma^{+}$ CO${}^{+}$ Vibrational Frequencies, Distortion Constants and Geometrical Parameters.

	Mode	$\omega_{1}$ ($a_{1}$)	$\nu_{1}$ ($a_{1}$)	B${}_{e}$	B${}_{0}$	B${}_{1}$	D${}_{e}$	H${}_{e}$	r${}_{0}$
	Units	cm${}^{-1}$	cm${}^{-1}$	MHz	MHz	MHz	kHz	mHz	Å
	Exp. ${}^{a}$		2183.9		59,270.5				
	F12-TZ	2213.3	2183.0	59,058	58,776	58,212	187.191	126.305	1.121
	CcCR	2224.6	2194.0	59,434	59,148	58,577	188.852	122.642	1.118
EOM-IP-CC3	TQcCT	2228.8	2198.3	59,405	59,122	58,557	187.860	130.221	1.118
	CcCT	2226.3	2196.2	59,355	59,073	58,509	187.810	131.241	1.119
EOM-IP-CCSDT-3	QZ	2182.6	2151.0	58,776	58,482	57,896	189.746	92.347	1.124
	5Z	2187.2	2155.6	58,883	58,590	58,004	189.990	94.210	1.123
	TQ5	2192.1	2160.3	58,995	58,702	58,115	190.213	94.982	1.122
	TQ5+cC	2201.0	2168.9	59,188	58,894	58,307	190.537	96.961	1.120
	TQcCT	2229.7	2199.2	59,412	59,130	58,565	187.787	130.683	1.118
	CcCT	2227.2	2197.1	59,363	59,081	58,518	187.731	131.768	1.118
EOM-IP-CCSDT	TQcC	2232.9	2202.5	59,447	59,166	58,602	187.578	132.036	1.118
	CcC	2230.8	2200.7	59,402	59,121	58,558	187.497	133.314	1.118

${}^{a}$ Refs. [17,18].

**Table 2:** $\tilde{A}$${}^{2}\Pi$ CO${}^{+}$ Vibrational Frequencies, Distortion Constants and Geometrical Parameters.

	Parameter	$\omega_{1}$ ($a_{1}$)	$\nu_{1}$ ($a_{1}$)	B${}_{e}$	B${}_{0}$	B${}_{1}$	D${}_{e}$	H${}_{e}$	r${}_{0}$	AEE
	Units	cm${}^{-1}$	cm${}^{-1}$	MHz	MHz	MHz	kHz	mHz	Å	cm${}^{-1}$
	Exp. ${}^{a}$		1534.9		47,649					20,733.3
	F12-TZ	1564.8	1538.7	47,469	47,186	46,618	194.456	25.181	1.252	20,147.8
	CcCR	1569.5	1543.7	47,745	47,461	46,893	196.682	37.944	1.248	20,420.7
	(T)+EOM/CcCR	1467.6	1425.2	47,042	46,676	45,944	215.167	−415.627	1.259	20,091.1
EOM-EE-CC3	TQcC	1496.2	1456.8	47,271	46,923	46,227	210.052	−305.464	1.255	18,394.2
	CcC	1495.2	1456.3	47,218	46,871	46,177	209.623	−299.783	1.256	18,344.4
EOM-IP-CC3	TQcCT	1582.3	1556.1	47,864	47,581	47,013	194.969	34.177	1.247	20,446.7
	CcCT	1581.3	1555.3	47,813	47,530	46,965	194.601	37.994	1.247	20,405.4
EOM-IP-CCSDT-3	QZ	1573.1	1546.6	47,541	47,258	46,692	193.297	26.122	1.251	20,568.8
	5Z	1576.1	1550.1	47,618	47,335	46,770	193.497	29.761	1.250	20,677.3
	TQ5	1579.0	1552.9	47,706	47,423	46,857	193.845	30.579	1.249	20,786.3
	TQ5+cC	1585.8	1559.2	47,881	47,596	47,028	194.301	29.645	1.246	21,008.8
	TQcCT	1581.9	1555.2	47,860	47,576	47,008	195.026	33.348	1.247	20,410.8
	CcCT	1580.9	1554.9	47,809	47,526	46,960	194.653	36.526	1.247	20,365.3
EOM-IP-CCSDT	TQcC	1583.4	1557.1	47,878	47,595	47,028	194.873	35.115	1.246	20,387.3
	CcC	1582.6	1556.7	47,831	47,548	46,984	194.481	38.936	1.247	20,356.7

${}^{a}$ Refs. [17,18].

**Table 3:** $\tilde{B}$${}^{2}\Sigma^{+}$ CO${}^{+}$ Vibrational Frequencies, Distortion Constants and Geometrical Parameters.

	Mode	$\omega_{1}$ ($a_{1}$)	$\nu_{1}$ ($a_{1}$)	B${}_{e}$	B${}_{0}$	B${}_{1}$	D${}_{e}$	H${}_{e}$	r${}_{0}$	AEE
	Units	cm${}^{-1}$	cm${}^{-1}$	MHz	MHz	MHz	kHz	mHz	Å	cm${}^{-1}$
	Exp. ${}^{a}$		1678.3		53,930					45,876.7
	(T)+EOM/CcCR	1212.1	1195.8	43,868	44,111	44,597	255.81	5.262	1.295	45,293.3
EOM-EE-CC3	TQcC	1808.2	1710.2	54,283	53,832	52,929	217.777	−0.670	1.172	42,534.7
	CcC	1806.0	1711.7	54,184	53,733	52,830	217.129	−0.673	1.173	44,460.8
EOM-IP-CC3	TQcCT	1843.7	1803.9	54,549	54,195	53,489	212.563	−0.154	1.168	45,229.5
	CcCT	1841.8	1802.3	54,489	54,136	53,430	212.305	−0.154	1.169	45,220.3
EOM-IP-CCSDT-3	QZ	1831.7	1787.8	54,343	53,974	53,237	212.921	−236.013	1.170	46,555.9
	5Z	1837.5	1793.3	54,444	54,077	53,343	212.789	−226.247	1.170	46,636.7
	TQ5	1843.0	1798.9	54,556	54,190	53,459	212.813	−218.012	1.168	46,707.4
	TQ5+cC	1856.1	1812.0	54,774	54,411	53,684	212.339	−201.694	1.166	46,725.4
	TQcCT	1844.7	1805.5	54,552	54,200	53,495	212.364	−0.149	1.168	45,199.8
	CcCT	1843.0	1803.6	54,493	54,141	53,437	212.084	−0.149	1.169	45,191.2
EOM-IP-CCSDT	TQcC	1852.4	1813.3	54,610	54,262	53,565	211.286	−0.131	1.167	45,187.6
	CcC	1841.3	1801.1	54,577	54,253	53,604	213.45	−0.002	1.167	45,180.8

${}^{a}$ Refs. [17,18].

**Table 4:** $\tilde{X}$${}^{2}B_{1}$ H${}_{2}$O${}^{+}$ Vibrational Frequencies in cm${}^{-1}$.

	Mode	$\omega_{1}$ (a${}_{1}$)	$\omega_{2}$ (a${}_{1}$)	$\omega_{3}$ (b${}_{2}$)	$\nu_{1}$ (a${}_{1}$)	$\nu_{2}$ (a${}_{1}$)	$\nu_{3}$ (b${}_{2}$)
	Description	Sym. Str.	Bend	Anti Sym. Str.	Sym. Str.	Bend	Anti Sym. Str.
	Exp. ${}^{a}$				3212.86		
	Exp. ${}^{b}$					1408.42	
	Exp. ${}^{c}$						3259.04
	Exp. ${}^{d}$				3267	1435	3299
	Prev theory ^e^	3389.7	1478.4	3440.9			
	F12-TZ	3383.8	1473.1	3441.1	3211.6	1408.4	3256.2
	CcCR	3389.4	1473.5	3446.9	3215.4	1408.3	3260.2
EOM-IP-CC3	TQcCT	3387.8	1469.0	3445.7	3209.2	1403.4	3255.5
	CcCT	3385.6	1468.3	3444.3	3209.9	1401.1	3256.8
EOM-IP-CCSDT-3	QZ	3429.1	1472.1	3373.2	3193.7	1407.2	3236.9
	5Z	3432.1	1471.1	3375.4	3200.9	1405.9	3245.0
	TQ5	3435.7	1470.8	3378.7	3202.3	1405.5	3246.7
	TQ5+cC	3440.3	1469.7	3383.2	3206.0	1404.3	3250.5
	TQcCT	3381.5	1468.2	3438.4	3197.7	1403.1	3242.7
	CcCT	3378.3	1467.3	3436.0	3202.6	1401.8	3248.2
EOM-IP-CCSDT	TQcC	3381.3	1468.2	3438.4	3202.8	1403.0	3248.0
	VPT2 CcC	3378.6	1467.4	3436.6	3203.0	1402.3	3248.7
	VCI CcC	3378.6	1467.4	3436.6	3202.1	1399.1	3248.8

${}^{a}$ Refs. [79,80], ${}^{b}$ Refs. [20,79,80,81]; ${}^{c}$ Refs. [20,36,37,82]; ${}^{d}$ Ref. [25]; ^e^ Ref. [38] CCSD(T)/aug-cc-pV5Z with additive corrections for core correlation; scalar relativistic effects and higher order correlation recovery effects.

**Table 5:** $\tilde{X}$${}^{2}B_{1}$ H${}_{2}$O${}^{+}$ Rotational Constants in MHz.

					EOM-IP-CC3		EOM-IP-CCSDT-3		EOM-IP-CCSDT	
Const.	Units	Exp. [83]	F12-TZ	CcCR	TQcCT	CcCT	TQcCT	CcCT	TQcC	CcC
A${}_{e}$	MHz		845,854	850,282	852,770	852,091	851,738	850,955	851,909	851,237
B${}_{e}$	MHz		376,983	377,740	377,246	376,888	376,938	376,518	376,901	376,508
C${}_{e}$	MHz		260,765	261,547	261,544	261,308	261,300	261,024	261,298	261,046
A${}_{0}$	MHz	870,580.8	870,304	875,321	878,528	877,794	877,314	876,429	877,501	876,766
B${}_{0}$	MHz	372,365.4	372,128	372,776	372,128	371,833	371,813	371,453	371,760	371,438
C${}_{0}$	MHz	253,880.4	253,591	254,321	254,272	254,071	254,017	253,770	254,007	253,792
A${}_{1}$	MHz	835,041.1	836,541	841,221	844,428	843,801	843,183	842,374	843,353	842,725
B${}_{1}$	MHz	367,803.7	367,340	367,948	367,192	366,947	366,858	366,543	366,793	366,525
C${}_{1}$	MHz	249,733.7	249,293	249,983	249,893	249,721	249,629	249,408	249,613	249,431
A${}_{2}$	MHz	1,001,285.4	971,991	978,731	983,370	982,335	981,829	980,613	982,069	981,019
B${}_{2}$	MHz	374,077.5	374,025	374,542	373,793	373,525	373,498	373,167	373,439	373,148
C${}_{2}$	MHz	249,275.7	249,083	249,795	249,751	249,553	249,488	249,244	249,478	249,266
A${}_{3}$	MHz	851,254.6	851,352	856,162	859,372	858,726	858,151	857,320	858,334	857,682
B${}_{3}$	MHz	365,511.7	364,952	365,549	364,797	364,554	364,470	364,156	364,404	364,138
C${}_{3}$	MHz	248,680.5	248,337	249,023	248,922	248,756	248,659	248,442	248,643	248,465

**Table 6:** $\tilde{A}$${}^{2}A_{1}$ H${}_{2}$O${}^{+}$ Vibrational Frequencies in cm${}^{-1}$.

	Mode	$\omega_{1}$(a${}_{g}$)	$\omega_{2}$(b${}_{2u}$)	$\omega_{3}$(b${}_{1u}$)	$\nu_{1}$(a${}_{g}$)	$\nu_{2}$(b${}_{2u}$)	$\nu_{3}$(b${}_{1u}$)	AEE
	Description	Sym. Str.	Bend	Anti Sym. Str.	Sym. Str.	Bend	Anti Sym. Str.	
	Exp. ${}^{a}$				3547 ± 16	876.8		
	Exp. ${}^{b}$				3153 ± 169	903 ± 80		
	Previous theory ${}^{c}$	3388 ${}^{c}$	403 ${}^{c}$	3624.3 ${}^{c}$				
	Previous theory ${}^{d}$							7886 ${}^{d}$
	F12-TZ	3387.1	392.5	3623.6	3228.9	1036.4	3416.9	8026.0
	CcCR	3393.3	405.0	3628.8	3232.1	995.0	3420.7	7951.0
	(T)+EOM/CcCR	3394.3	373.2	3629.8	3231.7	1056.6	3425.2	8051.4
EOM-IP-CC3	TQcCT	3385.6	430.0	3612.7	3224.5	689.4	3407.6	7057.6
	CcCT	3377.4	424.5	3606.1	3217.4	690.4	3401.9	7060.5
EOM-IP-CCSDT-3	QZ	3609.8	419.7	3380.4	3223.9	906.9	3404.1	7958.9
	5Z	3607.6	420.9	3377.8	3222.6	900.5	3404.8	7918.4
	TQ5	3608.9	424.4	3379.6	3224.3	912.9	3406.0	7892.9
	TQ5+cC	3613.4	427.1	3384.7	3227.2	888.9	3408.8	7817.2
	TQcCT	3385.5	428.7	3612.8	3228.0	1098.4	3406.7	7899.8
	CcCT	3377.5	423.5	3606.4	3222.2	1067.1	3405.4	7903.1
EOM-IP-CCSDT	TQcCT	3384.4	429.2	3612.0	3223.7	957.5	3407.0	7865.2
	VPT2 CcCT	3377.0	424.1	3606.4	3222.0	970.2	3405.3	7851.0
	VCI CcCT	3377.0	424.1	3606.4	3212.8	557.5	3404.8	7710.7

${}^{a}$ Ref. [20]; ${}^{b}$ Ref. [25]; ${}^{c}$ Ref. [38]; ${}^{d}$ Ref. [35].

**Table 7:** $\tilde{B}$${}^{2}B_{2}$ H${}_{2}$O${}^{+}$ Vibrational Frequencies in cm${}^{-1}$.

	Mode	$\omega_{1}$ ($a_{1}$)	$\omega_{2}$ ($a_{1}$)	$\omega_{3}$ ($b_{2}$)	$\nu_{1}$ ($a_{1}$)	$\nu_{2}$ ($a_{1}$)	$\nu_{3}$ ($b_{2}$)	AEE
	Description	Sym. Str.	Bend	Anti Sym. Str.	Sym. Str.	Bend	Anti Sym. Str.	
	Exp. ${}^{a}$				2968	1596		36,757 ± 12
	Exp. ${}^{b}$				2903 ± 80	1532 ± 80	2839 ± 56	
	Previous Theory ${}^{c}$	2613.9	1597.4	1945.1				
	(T)+EOM/CcCR	2617.8	1589.0	1958.3	2383.8	1467.0	1746.1	33,880.0
EOM-EE-CC3	TQcC	2618.7	1602.4	1964.8	2394.9	1480.4	1760.3	33,919.9
	CcC	2622.6	1603.6	1969.8	2398.1	1479.5	1764.1	33,881.7
EOM-IP-CC3	TQcCT	2627.6	1594.9	1978.8	2404.5	1479.7	1781.1	33,353.6
	CcCT	2631.5	1596.2	1984.0	2407.8	1477.4	1783.5	33,313.5
EOM-IP-CCSDT-3	QZ	2628.4	1608.1	1969.1	2406.1	1486.2	1766.5	33,917.5
	5Z	2627.0	1607.2	1967.3	2404.8	1486.5	1765.1	33,901.7
	TQ5	2630.7	1609.3	1970.4	2406.8	1488.7	1763.0	33,914.7
	TQ5+cC	2627.6	1608.2	1968.0	2405.2	1486.9	1764.4	33,967.5
	TQcCT	2626.9	1595.2	1978.1	2403.8	1478.8	1777.1	33,980.4
	CcCT	2630.8	1596.6	1983.2	2407.3	1477.1	1782.1	33,956.6
EOM-IP-CCSDT	TQcC	2628.3	1595.7	1980.2	2406.2	1480.2	1782.2	34,005.7
	VPT2 CcC	2632.3	1597.1	1985.1	2409.3	1478.9	1785.7	33,970.4
	VCI CcC	2632.3	1597.1	1985.1	2433.1	1561.7	1783.3	33,987.2

${}^{a}$ Ref. [20]; ${}^{b}$ Ref. [25]; ${}^{c}$ Ref. [38] CCSD(T)/aug-cc-pV5Z with additive corrections for core correlation; scalar relativistic effects and higher order correlation recovery effects.

**Table 8:** $\tilde{X}$${}^{2}B_{1}$ H${}_{2}$CO${}^{+}$ Vibrational Frequencies in cm${}^{-1}$.

					EOM-IP-CCSDT-3				
Mode	Description	Exp	F12-TZ	CcCR	QZ	TQ	TQ+cC	VPT2 TQcCT	VCI TQcCT
$\omega_{1}$ (a${}_{1}$)	Sym. C–H str.		2796.4	2797.7	2790.5	2785.9	2788.4	2805.6	2805.6
$\omega_{2}$ (a${}_{1}$)	O–C str.		1676.8	1683.2	1688.9	1697.1	1704.7	1677.2	1677.2
$\omega_{3}$ (a${}_{1}$)	H–C–O sym. bend		1257.9	1258.6	1253.7	1253.2	1253.6	1262.0	1262.0
$\omega_{4}$ (b${}_{1}$)	Out-of-plane bend		1062.1	1064.3	1063.6	1064.0	1064.9	1065.7	1065.7
$\omega_{5}$ (b${}_{2}$)	Anti sym. C–H str.		2904.3	2906.4	2892.7	2889.2	2890.7	2915.5	2915.5
$\omega_{6}$ (b${}_{2}$)	H–C–O anti sym. bend		842.8	844.2	841.0	841.1	842.1	847.9	847.9
$\nu_{1}$ (a${}_{1}$)	Sym. C–H str.	2580 ± 4 ${}^{b,c,d}$	2616.6	2612.1	2608.9	2603.0	2604.2	2624.2	2624.6
$\nu_{2}$ (a${}_{1}$)	O–C str.	1675 ± 4 ${}^{c,d}$	1681.9	1669.9	1684.0	1690.1	1696.7	1677.3	1675.9
$\nu_{3}$ (a${}_{1}$)	H–C–O sym. bend	1210 ± 4 ${}^{a,b,c,d}$	1209.9	1196.3	1202.3	1200.4	1200.9	1210.8	1217.4
$\nu_{4}$ (b${}_{1}$)	Out-of-plane bend	1036 ± 4 ^e^	1013.6	1007.9	1038.9	1036.0	1036.7	1039.2	1035.1
$\nu_{5}$ (b${}_{2}$)	Anti sym. C–H str.	2718.24 ± ${}^{a}$	2700.4	2695.1	2688.3	2682.0	2682.4	2710.4	2715.5
$\nu_{6}$ (b${}_{2}$)	H–C–O anti sym. bend	823.7 ± 0.3 ^e^	825.0	762.9	819.3	817.7	819.0	824.4	820.0

${}^{a}$ Ref. [84], ${}^{b}$ Ref. [28], ${}^{c}$ Ref. [29], ${}^{d}$ Ref. [30], ^e^ Ref. [85].

**Table 9:** $\tilde{A}$${}^{2}B_{1}$ H${}_{2}$CO${}^{+}$ Vibrational Frequencies in cm${}^{-1}$.

				EOM-IP-CCSDT-3				
	Description	Exp	(T)+EOM/CcCR	QZ	TQ	TQ+cC	VPT2 TQcCT	VCI TQcCT
$\omega_{1}$ (a${}_{1}$)	Sym. C–H str.		3041.9	3041.5	3086.0	3041.9	3041.5	3041.5
$\omega_{2}$ (a${}_{1}$)	O–C str.		1510.3	1530.3	1556.5	1510.3	1530.3	1530.3
$\omega_{3}$ (a${}_{1}$)	H–C–O sym. bend		1257.2	1290.9	1291.6	1257.2	1290.9	1290.9
$\omega_{4}$ (b${}_{1}$)	Out-of-plane bend		1196.0	1210.5	1235.5	1196.0	1210.5	1210.5
$\omega_{5}$ (b${}_{2}$)	Anti sym. C–H str.		3196.7	3199.0	3238.6	3196.7	3199.0	3199.0
$\omega_{6}$ (b${}_{2}$)	H–C–O anti sym. bend		1162.4	1177.7	1195.7	1162.4	1177.7	1177.7
$\nu_{1}$ (a${}_{1}$)	Sym. C–H str.		2870.9	2876.8	2928.1	2870.9	2876.8	2889.1
$\nu_{2}$ (a${}_{1}$)	O–C str.	1488 ± 4 ${}^{a,b}$	1470.1	1489.3	1516.0	1470.1	1489.3	1505.7
$\nu_{3}$ (a${}_{1}$)	H–C–O sym. bend	1250 ± 4 ${}^{a,b,c}$	1226.4	1262.0	1260.7	1226.4	1262.0	1263.2
$\nu_{4}$ (b${}_{1}$)	Out-of-plane bend		1179.0	1191.9	1218.3	1179.0	1191.9	1192.2
$\nu_{5}$ (b${}_{2}$)	Anti sym. C–H str.		3052.7	3050.2	3096.2	3052.7	3050.2	3050.9
$\nu_{6}$ (b${}_{2}$)	H–C–O anti sym. bend		1137.2	1154.9	1168.7	1137.2	1154.9	1152.7
AEE		25,929 ± 5 ${}^{a,b,c}$	25,248.0	25,748.5	25,935.6	25,975.0	26,469.6	26,471.7

${}^{a}$ Ref. [29], ${}^{b}$ Ref. [30], ${}^{c}$ Ref. [28].

**Table 10:** $\tilde{B}$${}^{2}A_{1}$ H${}_{2}$CO${}^{+}$ Vibrational Frequencies in cm${}^{-1}$.

				EOM-IP-CCSDT-3				
	Description	Exp.	(T)+EOM/CcCR	QZ	TQ	TQcC	VPT2 TQcCT	VCI TQcCT
$\omega_{1}$ (a${}_{1}$)	Sym. C–H str.		2897.7	2865.1	2865.6	2871.1	2877.0	2877.0
$\omega_{2}$ (a${}_{1}$)	O–C str.		1462.0	1387.8	1390.3	1394.0	1397.4	1397.4
$\omega_{3}$ (a${}_{1}$)	H–C–O sym. bend		1390.8	1350.2	1355.6	1361.2	1371.9	1371.9
$\omega_{4}$ (b${}_{1}$)	Out-of-plane bend		1248.2	1236.0	1238.9	1242.0	1245.6	1245.6
$\omega_{5}$ (b${}_{2}$)	Anti sym. C–H str.		3061.8	3012.8	3014.0	3019.2	3031.4	3031.4
$\omega_{6}$ (b${}_{2}$)	H–C–O anti sym. bend		1234.4	1213.1	1218.0	1221.8	1225.4	1225.4
$\nu_{1}$ (a${}_{1}$)	Sym. C–H str.		2764.6	2706.3	2710.8	2718.6	2733.8	2653.8
$\nu_{2}$ (a${}_{1}$)	O–C str.	1304 ± 4 ${}^{a}$	1430.5	1348.5	1350.9	1354.6	1360.0	1374.8
$\nu_{3}$ (a${}_{1}$)	H–C–O sym. bend		1352.8	1304.6	1309.6	1314.4	1326.4	1346.3
$\nu_{4}$ (b${}_{1}$)	Out-of-plane bend		1242.1	1213.5	1217.7	1218.9	1221.5	1213.4
$\nu_{5}$ (b${}_{2}$)	Anti sym. C–H str.		2858.8	2781.1	2780.3	2784.7	2803.1	2809.9
$\nu_{6}$ (b${}_{2}$)	H–C–O anti sym. bend		1209.4	1178.2	1182.1	1185.6	1192.6	1189.7
AEE		39,928 ± 6 ${}^{a}$	40,883.0	39,758.9	39,864.8	39,960.3	39,881.8	39,887.6

${}^{a}$ Refs. [28,29,30].

## Data Availability

All required data are availble in the manuscript.

## Abbreviations

The following abbreviations are used in this manuscript:

CCSD(T)	Coupled cluster singles and doubles and perturbative triples
CCSDT	Coupled cluster singles doubles and triples
EOM-EE	Equation of motion excitation energy
EOM-IP	Equation of motion ionization potential
CBS	Complete basis set extrapolation
CcCT	Three-point CBS with core correlation and higher order correlation corrections
QFF	Quartic force field
MAE	Mean absolute error
MAD	Mean absolute difference
F12-TZ	QFF approach using CCSD(T)-F12 explicitly correlated methods
CcCR	QFF using three point CBS, core correlation and scalar relativistic corrections
(T)+EOM	Electronically excited state QFF using CCSD(T) and EOM-CCSD energies

## Appendix A. Symmetry Internal Coordinate Schemes

The SIC schemes for each species are as follows. For the $\tilde{X}$ ${}^{2}$B${}_{1}$ and $\tilde{B}$ ${}^{2}$B${}_{2}$ states of H${}_{2}$O${}^{+}$, a scheme of 69 points uses the following equations:

(A1) $$\begin{array}{cc} S_{1}\left(a_{1}\right)= & \frac{1}{\sqrt{2}}\left[r\left(H_{1}-O\right)+r\left(H_{2}-O\right)\right] \end{array}$$

(A2) $$\begin{array}{cc} S_{2}\left(a_{1}\right)= & ∠(H_{1}-O-H_{2}) \end{array}$$

(A3) $$\begin{array}{cc} S_{3}\left(b_{2}\right)= & \frac{1}{\sqrt{2}}\left[r\left(H_{1}-O\right)-r\left(H_{2}-O\right)\right] \end{array}$$

For the $\tilde{A}$ ${}^{2}$A${}_{1}$ state, which is linear in its equilibrium configuration, the following scheme is used for 57 symmetry-unique points:

(A4) $$\begin{array}{cc} S_{1}\left(a_{g}\right)= & \frac{1}{\sqrt{2}}\left[r\left(H_{1}-O\right)+r\left(H_{2}-O\right)\right] \end{array}$$

(A5) $$\begin{array}{cc} S_{2}\left(b_{1u}\right)= & \frac{1}{\sqrt{2}}\left[r\left(H_{1}-O\right)-r\left(H_{2}-O\right)\right] \end{array}$$

(A6) $$\begin{array}{cc} S_{3}\left(b_{2u}\right)= & ∠{\left(H_{1}-O-H_{2}\right)}_{x} \end{array}$$

(A7) $$\begin{array}{cc} S_{4}\left(b_{3u}\right)= & ∠{\left(H_{1}-O-H_{2}\right)}_{y} \end{array}$$

For all three states of H${}_{2}$CO${}^{+}$, the following scheme consisting of 413 points is used:

(A8) $$\begin{array}{cc} S_{1}\left(a_{1}\right)= & O-C \end{array}$$

(A9) $$\begin{array}{cc} S_{2}\left(a_{1}\right)= & \frac{1}{\sqrt{2}}\left[r\left(H_{1}-C\right)+r\left(H_{2}-C\right)\right] \end{array}$$

(A10) $$\begin{array}{cc} S_{3}\left(a_{1}\right)= & \frac{1}{\sqrt{2}}\left[∠\left(H_{1}-C-O\right)+∠\left(H_{2}-C-O\right)\right] \end{array}$$

(A11) $$\begin{array}{cc} S_{4}\left(b_{2}\right)= & \frac{1}{\sqrt{2}}\left[r\left(H_{1}-C\right)-r\left(H_{2}-C\right)\right] \end{array}$$

(A12) $$\begin{array}{cc} S_{5}\left(b_{2}\right)= & \frac{1}{\sqrt{2}}\left[∠\left(H_{1}-C-O\right)-∠\left(H_{2}-C-O\right)\right] \end{array}$$

(A13) $$\begin{array}{cc} S_{6}\left(b_{1}\right)= & OPB(O-C-H_{1}-H_{2}) \end{array}$$

All three states of CO${}^{+}$ use a trivial one-dimensional scheme displacing along the C–O bond length coordinate.

## Appendix B. Rotational Constants, Distortion Constants and Geometrical Parameters

**Table A1:** $\tilde{X}$${}^{2}B_{1}$ H${}_{2}$O${}^{+}$ Geometrical Parameters and Quartic & Sextic Distortion Constants.

				EOM-IP-CC3		EOM-IP-CCSDT-3		EOM-IP-CCSDT	
Const.	Units	F12-TZ	CcCR	TQcCT	CcCT	TQcCT	CcCT	TQcC	CcC
r${}_{0}$(H–O)	Å	0.999	0.998	0.998	0.998	0.995	0.999	0.998	0.999
${∠}_{0}$(H${}_{1}$–O–H${}_{2}$)	degrees	109.372	109.458	109.573	109.577	109.708	109.567	109.570	109.577
$\Delta_{J}$	MHz	28.045	28.163	28.19	28.116	28.185	28.106	28.18	28.1
$\Delta_{K}$	MHz	1.023	1.038	1.053	1.052	1.05	1.049	1.051	1.05
$\Delta_{JK}$	MHz	−129.999	−131.243	−132.631	−132.188	−132.166	−131.659	−132.174	−131.678
$\delta_{j}$	MHz	10.574	10.613	10.623	10.591	10.617	10.582	10.614	10.579
$\delta_{k}$	MHz	21.091	21.313	21.48	21.416	21.544	21.486	21.561	21.503
$\Phi_{J}$	kHz	9.068	9.089	9.092	9.056	9.093	9.055	9.087	9.049
$\Phi_{K}$	MHz	4.039	4.152	4.268	4.258	4.25	4.24	4.254	4.245
$\Phi_{JK}$	kHz	−58.887	−58.859	−58.893	−58.714	−58.916	−58.744	−58.883	−58.697
$\Phi_{KJ}$	kHz	−426.562	−439.749	−452.737	−450.271	−450.597	−448.009	−451.065	−448.474
$\phi_{j}$	kHz	4.47	4.48	4.482	4.464	4.483	4.464	4.48	4.461
$\phi_{jk}$	kHz	−9.131	−9.129	−9.191	−9.15	−9.172	−9.13	−9.169	−9.12
$\phi_{k}$	kHz	495.786	504.168	513.004	512.218	512.173	511.468	512.478	511.843

**Table A2:** $\tilde{A}$${}^{2}A_{1}$ H${}_{2}$O${}^{+}$ Rotational Constants, Distortion Constants and Geometrical Parameters.

					EOM-IP-CC3		EOM-IP-CCSDT-3		EOM-IP-CCSDT	
Const.	Units	F12-TZ	CcCR	(T)+EOM/CcCR	TQcCT	CcCT	TQcCT	CcCT	TQcCT	CcCT
B${}_{e}$	MHz	256,623	257,362	257,404	256,998	256,669	257,004	256,683	256,976	256,683
B${}_{0}$	MHz	257,183	257,675	258,240	256,974	256,756	256,989	256,782	256,966	256,772
B${}_{1}$	MHz	251,201	251,670	252,227	250,913	250,708	250,926	250,732	250,901	250,720
B${}_{2}$	MHz	263,302	263,588	264,681	262,595	262,476	262,621	262,516	262,604	262,498
B${}_{3}$	MHz	252,044	252,480	253,045	251,748	251,539	251,759	251,560	251,737	251,548
r${}_{0}$(H–O)	Å	0.988	0.987	0.987	0.988	0.988	0.988	0.988	0.988	0.988
D${}_{e}$	MHz	6.558	6.59	6.59	6.592	6.599	6.593	6.6	6.595	6.601
H${}_{e}$	Hz	114.068	113.27	113.276	112.027	112.767	111.924	112.606	112.042	112.608

**Table A3:** $\tilde{B}$${}^{2}B_{1}$ H${}_{2}$O${}^{+}$ Rotational Constants (MHz).

		EOM-EE-CC3		EOM-IP-CC3		EOM-IP-CCSDT-3		EOM-IP-CCSDT	
Const.	(T)+EOM/CcCR	TQcC	CcC	TQcCT	CcCT	TQcCT	CcCT	TQcC	CcC
A${}_{e}$	856,740	854,842		854,876	854,475	854,570	854,171	854,513	854,046
B${}_{e}$	290,373	291,052		291,429	291,253	291,433	291,261	291,532	291,395
C${}_{e}$	216,870	217,126		217,338	217,214	217,320	217,199	217,372	217,265
A${}_{0}$	842,278	840,270		841,290	841,296	840,911	840,922	840,795	840,718
B${}_{0}$	287,185	287,966		288,324	288,128	288,341	288,148	288,472	288,315
C${}_{0}$	208,698	209,019		209,272	209,179	209,258	209,166	209,323	209,245
A${}_{1}$	831,448	828,877		830,261	830,478	829,862	830,084	829,773	829,905
B${}_{1}$	280,364	281,231		281,624	281,434	281,641	281,453	281,775	281,618
C${}_{1}$	204,616	204,926		205,221	205,144	205,204	205,128	205,272	205,205
A${}_{2}$	826,003	824,940		826,033	826,413	825,656	826,034	825,401	825,723
B${}_{2}$	294,686	295,397		295,777	295,541	295,800	295,569	295,981	295,783
C${}_{2}$	190,296	190,199		191,267	191,351	191,212	191,298	191,344	191,424
A${}_{3}$	840,484	837,876		840,434	840,666	839,922	840,177	839,798	839,896
B${}_{3}$	279,904	280,878		281,137	280,940	281,176	280,977	281,316	281,162
C${}_{3}$	215,039	215,912		215,392	215,166	215,428	215,202	215,452	215,257

**Table A4:** $\tilde{B}$${}^{2}B_{1}$ H${}_{2}$O${}^{+}$ Distortion Constants and Geometrical Parameters.

			EOM-EE-CC3		EOM-IP-CC3		EOM-IP-CCSDT-3		EOM-IP-CCSDT	
Const.	Units	(T)+EOM/CcCR	TQcC	CcC	TQcCT	CcCT	TQcCT	CcCT	TQcC	CcC
r${}_{0}$(H–O)	Å	1.125	1.124		1.123	1.124	1.123	1.124	1.123	1.124
${∠}_{0}$(H${}_{1}$–O–H${}_{2}$)	degrees	57.501	57.611		57.642	57.638	57.651	57.648	57.660	57.662
$\Delta_{J}$	MHz	18.552	18.313		18.399	18.318	18.391	18.311	18.382	18.305
$\Delta_{K}$	MHz	713.318	703.328		712.235	710.605	711.331	709.702	711.308	709.531
$\Delta_{JK}$	MHz	22.464	25.023		21.547	21.004	21.754	21.213	21.582	21.093
$\delta_{j}$	MHz	5.576	5.484		5.54	5.517	5.536	5.514	5.535	5.514
$\delta_{k}$	MHz	70.21	69.475		68.705	68.245	68.704	68.246	68.577	68.138
$\Phi_{J}$	kHz	2.955	2.894		2.922	2.899	2.922	2.899	2.929	2.907
$\Phi_{K}$	MHz	1.094	1.09		1.097	1.096	1.097	1.095	1.095	1.094
$\Phi_{JK}$	kHz	−14.575	−13.396		−14.111	−14.095	−14.027	−14.036	−14.136	−14.096
$\Phi_{KJ}$	kHz	−9.603	−12.572		−7.459	−6.991	−7.955	−7.334	−7.311	−7.158
$\phi_{j}$	kHz	1.445	1.407		1.422	1.411	1.422	1.411	1.425	1.414
$\phi_{jk}$	kHz	1.062	1.552		1.043	0.973	1.098	1.018	1.106	1.045
$\phi_{k}$	kHz	758.931	737.041		740.336	733.847	739.376	732.933	738.163	731.694

**Table A5:** $\tilde{X}$${}^{2}B_{1}$ H${}_{2}$CO${}^{+}$ Rotational Constants.

Const.	F12-TZ	CcCR	EOM-IP-CCSDT-3/TQcCT
A${}_{e}$	266,451	267,249	267,221
B${}_{e}$	40,055	40,270	40,131
C${}_{e}$	34,820	34,997	34,891
A${}_{0}$	265,457	266,359	266,229
B${}_{0}$	40,053	40,266	40,117
C${}_{0}$	34,611	34,786	34,672
A${}_{1}$	260,866	261,828	261,575
B${}_{1}$	40,070	40,283	40,129
C${}_{1}$	34,550	34,725	34,608
A${}_{2}$	265,145	266,039	265,965
B${}_{2}$	39,673	39,885	39,734
C${}_{2}$	34,301	34,474	34,358
A${}_{3}$	269,591	270,557	270,364
B${}_{3}$	40,135	40,347	40,198
C${}_{3}$	34,560	34,734	34,621
A${}_{4}$	274,921	275,901	275,876
B${}_{4}$	40,041	40,253	40,101
C${}_{4}$	34,708	34,884	34,771
A${}_{5}$	262,537	263,492	263,246
B${}_{5}$	40,043	40,255	40,102
C${}_{6}$	34,568	34,743	34,625
A${}_{6}$	257,693	258,557	258,366
B${}_{6}$	40,349	40,563	40,406
C${}_{6}$	34,559	34,732	34,616

**Table A6:** $\tilde{X}$${}^{2}B_{1}$ H${}_{2}$CO${}^{+}$ Distortion Constants and Geometrical Parameters.

Const.	Units	F12-TZ	CcCR	EOM-IP-CCSDT-3/TQcCT
r${}_{0}$(O–C)	Å	1.194	1.190	1.193
r${}_{0}$(C–H)	Å	1.115	1.113	1.113
r${}_{0}$(C–O–H)	degrees	119.510	119.519	119.515
$\Delta_{J}$	MHz	86.027	86.811	86.876
$\Delta_{K}$	MHz	17.798	17.944	17.934
$\Delta_{JK}$	MHz	2.948	2.973	2.925
$\delta_{j}$	MHz	12.898	13.056	13.024
$\delta_{k}$	MHz	1.827	1.844	1.82
$\Phi_{J}$	kHz	138.483	142.723	137.905
$\Phi_{K}$	MHz	5.438	5.532	5.473
$\Phi_{JK}$	kHz	140.429	142.339	137.736
$\Phi_{KJ}$	kHz	−381.316	−388.997	−366.657
$\phi_{j}$	kHz	76.638	78.37	76.936
$\phi_{jk}$	kHz	72.63	73.622	71.246
$\phi_{k}$	kHz	3.8	3.844	3.772

**Table A7:** $\tilde{A}$${}^{2}B_{2}$ H${}_{2}$CO${}^{+}$ Rotational Constants MHz.

Const.	Units	(T)+EOM/CcCR	EOM-IP-CCSDT-3/TQcCT
A${}_{e}$	MHz	269,758	270,948
B${}_{e}$	MHz	32,266	32,886
C${}_{e}$	MHz	28,818	29,326
A${}_{0}$	MHz	266,957	267,989
B${}_{0}$	MHz	32,035	32,673
C${}_{0}$	MHz	28,498	29,016
A${}_{1}$	MHz	262,367	263,253
B${}_{1}$	MHz	32,002	32,642
C${}_{1}$	MHz	28,423	28,938
A${}_{2}$	MHz	268,992	269,927
B${}_{2}$	MHz	32,296	32,936
C${}_{2}$	MHz	28,361	28,873
A${}_{3}$	MHz	266,655	267,812
B${}_{3}$	MHz	31,730	32,393
C${}_{3}$	MHz	28,228	28,766
A${}_{4}$	MHz	263,372	264,300
B${}_{4}$	MHz	31,693	32,335
C${}_{4}$	MHz	28,536	29,061
A${}_{5}$	MHz	263,939	264,868
B${}_{5}$	MHz	31,987	32,624
C${}_{5}$	MHz	28,441	28,956
A${}_{6}$	MHz	270,816	271,856
B${}_{6}$	MHz	32,037	32,681
C${}_{6}$	MHz	28,360	28,879

**Table A8:** $\tilde{A}$${}^{2}B_{2}$ H${}_{2}$CO${}^{+}$ Distortion Constants and Geometrical Parameters.

Const.	Units	(T)+EOM/CcCR	EOM-IP-CCSDT-3/TQcCT
r${}_{0}$(O–C)	Å	1.351	1.338
r${}_{0}$(C–H)	Å	1.096	1.094
r${}_{0}$(C–O–H)	degrees	118.392	118.411
$\Delta_{J}$	kHz	77.129	77.161
$\Delta_{K}$	MHz	15.551	15.589
$\Delta_{JK}$	MHz	1.181	1.191
$\delta_{j}$	kHz	8.547	8.702
$\delta_{k}$	kHz	789.912	801.015
$\Phi_{J}$	mHz	−5.697	21.892
$\Phi_{K}$	kHz	2.94	2.914
$\Phi_{JK}$	Hz	19.343	20.026
$\Phi_{KJ}$	Hz	2.044	1.985
$\phi_{j}$	mHz	22.822	27.041
$\phi_{jk}$	Hz	10.187	10.576
$\phi_{k}$	kHz	1.07	1.073

**Table A9:** $\tilde{B}$${}^{2}A_{1}$ H${}_{2}$CO${}^{+}$ Rotational Constants MHz.

Const.	Units	(T)+EOM/CcCR	EOM-IP-CCSDT-3/TQcCT
A${}_{e}$	MHz	245,655.9	242,934.4
B${}_{e}$	MHz	36,911.9	36,487.7
C${}_{e}$	MHz	32,090.1	31,723.1
A${}_{0}$	MHz	241,710.3	238,804.3
B${}_{0}$	MHz	36,669.8	36,214.1
C${}_{0}$	MHz	31,712	31,319.6
A${}_{1}$	MHz	236,953.5	233,905.8
B${}_{1}$	MHz	36,679.8	36,239.7
C${}_{1}$	MHz	31,648.9	31,266
A${}_{2}$	MHz	240,867.6	239,228.5
B${}_{2}$	MHz	36,347.2	36,092.6
C${}_{2}$	MHz	31,506.7	31,149.6
A${}_{3}$	MHz	243,348.2	239,086.7
B${}_{3}$	MHz	36,628.6	35,890.9
C${}_{3}$	MHz	31,582.5	31,060.7
A${}_{4}$	MHz	238,071.2	235,154.9
B${}_{4}$	MHz	36,574	36,131.8
C${}_{4}$	MHz	31,699.5	31,295.9
A${}_{5}$	MHz	238,231.4	235,191
B${}_{5}$	MHz	36,655.2	36,209.7
C${}_{5}$	MHz	31,652.7	31,265.4
A${}_{6}$	MHz	244,899	241,998.8
B${}_{6}$	MHz	36,648.9	36,171.6
C${}_{6}$	MHz	31,426.7	31,074.4

**Table A10:** $\tilde{B}$${}^{2}A_{1}$ H${}_{2}$CO${}^{+}$ Distortion Constants and Geometrical Parameters.

Const.	Units	(T)+EOM/CcCR	EOM-IP-CCSDT-3/TQcCT
r${}_{0}$(O–C)	Å	1.275	1.285
r${}_{0}$(C–H)	Å	1.100	1.102
r${}_{0}$(C–O–H)	degrees	113.349	112.813
$\Delta_{J}$	kHz	83.017	88.493
$\Delta_{K}$	MHz	11.061	10.623
$\Delta_{JK}$	MHz	1.375	1.36
$\delta_{j}$	kHz	10.874	11.545
$\delta_{k}$	kHz	845.975	844.286
$\Phi_{J}$	mHz	46.056	−9.671
$\Phi_{K}$	kHz	1.651	1.515
$\Phi_{JK}$	Hz	19.308	18.596
$\Phi_{KJ}$	Hz	70.135	81.113
$\phi_{j}$	mHz	37.114	29.883
$\phi_{jk}$	Hz	10.652	10.09
$\phi_{k}$	Hz	855.801	846.142
